# Metabolic regulation of intestinal homeostasis: molecular and cellular mechanisms and diseases

**DOI:** 10.1002/mco2.776

**Published:** 2024-10-25

**Authors:** Ruolan Zhang, Ansu Perekatt, Lei Chen

**Affiliations:** ^1^ School of Life Science and Technology, Key Laboratory of Developmental Genes and Human Disease Southeast University Nanjing China; ^2^ Department of Chemistry and Chemical Biology Stevens Institute of Technology Hoboken New Jersey USA; ^3^ Institute of Microphysiological Systems Southeast University Nanjing China

**Keywords:** gut microbiota, intestinal homeostasis, intestinal stem cell, metabolite, nutrient metabolism

## Abstract

Metabolism serves not only as the organism's energy source but also yields metabolites crucial for maintaining tissue homeostasis and overall health. Intestinal stem cells (ISCs) maintain intestinal homeostasis through continuous self‐renewal and differentiation divisions. The intricate relationship between metabolic pathways and intestinal homeostasis underscores their crucial interplay. Metabolic pathways have been shown to directly regulate ISC self‐renewal and influence ISC fate decisions under homeostatic conditions, but the cellular and molecular mechanisms remain incompletely understood. Understanding the intricate involvement of various pathways in maintaining intestinal homeostasis holds promise for devising innovative strategies to address intestinal diseases. Here, we provide a comprehensive review of recent advances in the regulation of intestinal homeostasis. We describe the regulation of intestinal homeostasis from multiple perspectives, including the regulation of intestinal epithelial cells, the regulation of the tissue microenvironment, and the key role of nutrient metabolism. We highlight the regulation of intestinal homeostasis and ISC by nutrient metabolism. This review provides a multifaceted perspective on how intestinal homeostasis is regulated and provides ideas for intestinal diseases and repair of intestinal damage.

## Graphical abstract

The maintenance of intestinal homeostasis requires interactions between intestinal epithelium and microenvironment. The intricate relationship between metabolic pathways and intestinal homeostasis underscores their crucial interplay.

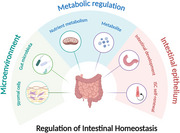

## 1 INTRODUCTION

The small intestine serves as the primary organ for food digestion and nutrient absorption, functioning as a critical immune barrier as well. Intestinal epithelial cells, known for their rapid turnover, undergo a complete renewal every 3–5 days.^1^, ^2^ However, this delicate balance can be disrupted by various internal and external stimuli, including mechanical stress, dysbiosis of the gut microbiota, and exposure to radiotherapy, chemotherapy, and inflammatory bowel diseases (IBDs). Such disruptions lead to compromised intestinal homeostasis, resulting in damage to intestinal epithelial cells and a decline in overall intestinal function.^3^, ^4^

Intestinal stem cells (ISCs) play a central role in maintaining intestinal homeostasis. The successful culture of small intestinal organoids in vitro and the use of transgenic mouse models contribute significantly to our understanding of ISC self‐renewal and differentiation. Digestive diseases are increasingly prevalent in recent years, and China may become the nation with the highest number of IBD cases globally.^5^, ^6^ Consequently, research efforts are increasingly focused on strategies to maintain and restore intestinal homeostasis.

ISCs exhibit remarkable plasticity, with the ability to self‐renew and differentiate into the various cell types necessary for normal intestinal function. The balance between ISC self‐renewal and differentiation is meticulously regulated by multiple signaling pathways, enabling ISCs to proliferate and give rise to specialized cell lineages. In this review, we provide a comprehensive overview of recent development in the regulation of intestinal homeostasis. We explore the status of the intestine and the properties of stem cells under various conditions, including homeostasis, injury, and aging. Additionally, we present the regulation of intestinal homeostasis from multiple perspectives, including the regulation of intestinal epithelium, microenvironment, and metabolic regulation. We also discuss the cellular and molecular mechanisms that underpin intestinal homeostasis, with a particular focus on the impact of metabolic processes within the intestine. Finally, we underscore the significance of nutrient metabolism in intestinal function, suggesting new avenues for metabolic research that may inform the treatment of intestinal diseases and facilitate the repair of intestinal damage.

## 2 CHARACTERISTICS OF THE INTESTINE

### 2.1 Characteristics of the intestine under homeostasis

**FIGURE 1:**
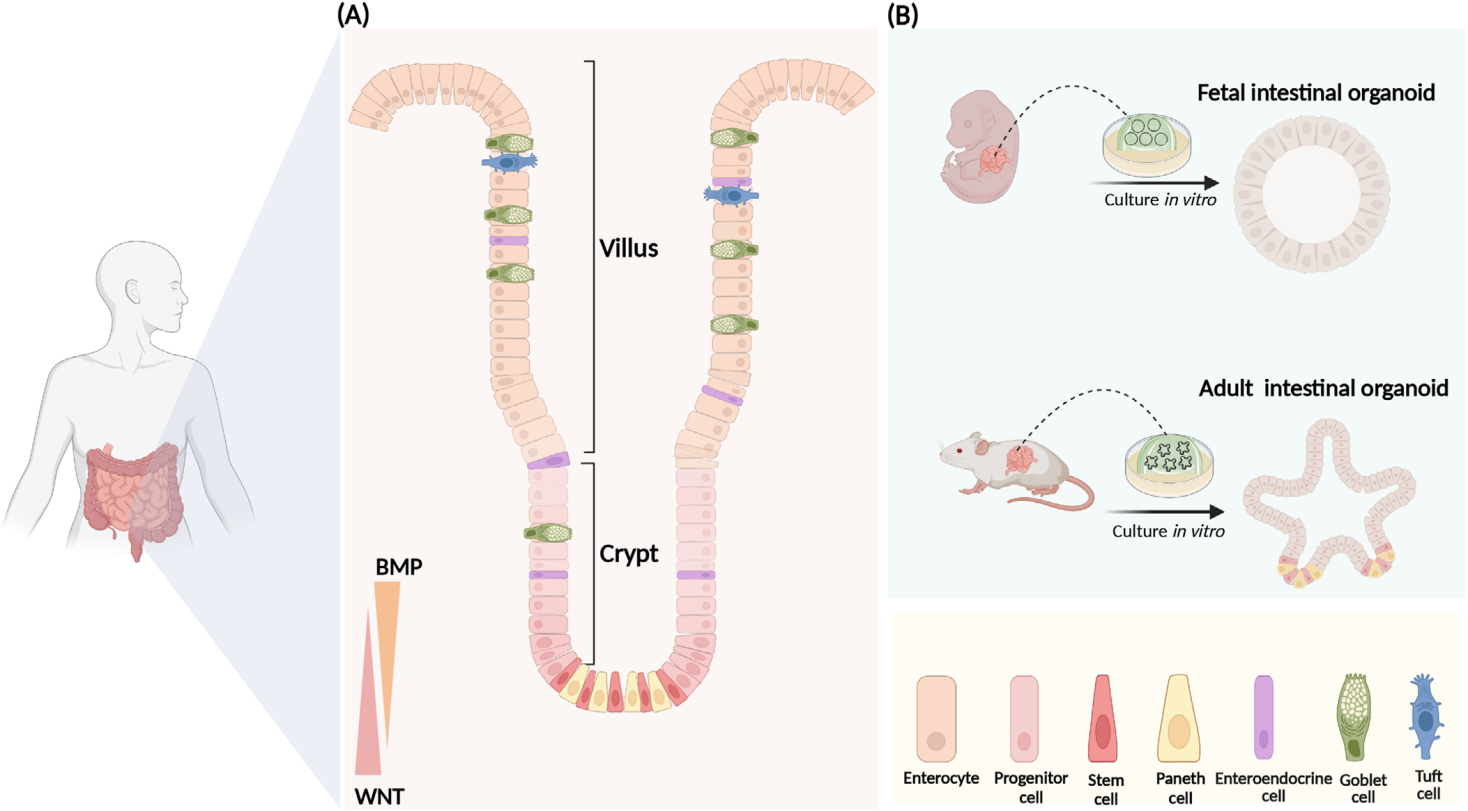
Epithelial and organoid structures of the small intestine. (A) The small intestinal epithelium is organized in units of crypts (proliferation zone) and villi (differentiation zone). Crypts are located at the bottom of the villi, and at the base of each crypt are wedge‐shaped crypt base columnar cells, also known as intestinal stem cells. (B) Small intestinal organoids can be categorized into fetal organoids and adult organoids according to their origin. Their transcriptomes and morphology display notable differences. BMP, bone morphogenetic protein; WNT, wingless‐related integration site.

The small intestine is the fastest renewing tissue in the adult body. The small intestine contains a significant number of immune cells and serves as a crucial immunological barrier in the body. In response to internal and external environmental stimuli, the intestinal epithelium is continuously resupplied by self‐renewal and migratory differentiation of stem cells to maintain intestinal homeostasis.^1^, ^7^, ^8^ The intestinal epithelium is lined by finger‐like structures called villus (Figure 1), which contain absorptive and secretory cells.^9^ Absorptive cells are responsible for nutrient and water uptake, while secretory cells include goblet cells, which secrete mucus; enteric endocrine cells, which produce hormones; and Paneth cells, which release antimicrobial peptides.^10^ These cells play a role in regulating intestinal immunity and other physiological functions.^1^, ^2^, ^9^ Lieberkühn crypts reside at the bottom of the villus, there are wedge‐shaped crypt base columnar (CBC) cells, which are also referred to as ISCs (Figure 1). Self‐renewal and differentiation of leucine‐rich repeat‐containing G‐protein‐coupled receptor 5 (Lgr5^+^) ISCs are critical for maintaining a stable turnover of intestinal epithelial cells.^10^, ^11^, ^12^ Lgr5^+^ ISCs located at the base of the crypts are widely regarded as the primary agents of intestinal epithelial renewal. There is also a type of reserve stem cells at the +4 position of the crypt, and these cells take over stem cell functions after the loss of Lgr5^+^ ISCs.^13^ Recent studies have shown that the main source of Lgr5^+^ ISC is in the upper crypts. Researchers have found a type of progenitor cell that expresses fibroblast growth factor‐binding protein 1 (*Fgfbp1*) in the upper crypts. These *Fgfbp1^+^* cells, distinct from the previously identified +4 cells, possess the capability to proliferate bidirectionally. Under homeostatic conditions, *Fgfbp1* ^+^ cells can migrate upward to differentiate into mature cells or migrate downward to form Lgr5^+^ ISCs.^14^ Intestinal homeostasis is coregulated by multiple signaling pathways. The wingless‐related integration site (WNT)/β‐catenin signaling is highly enriched in crypts and drives ISC self‐renewal. The bone morphogenetic protein (BMP) signaling pathway promotes differentiation of ISCs into mature cells. When intestinal epithelial cells are damaged, the Yes‐associated protein (YAP) signaling pathway induces intestinal cell reprogramming, enabling rapid repair.^15^, ^16^, ^17^, ^18^, ^19^ Small intestinal organoids have emerged in recent years as powerful tools for studying intestinal development and disease. Intestinal organoids are three‐dimensional (3D) mini‐intestines with intact crypts and villus structural domains. These organoids can be cultured from a crypt or a single Lgr5^+^ ISC.^20^

The intestine initially arises from endodermal cells.^21^ At days 8–9.5 of embryonic development (E8–E9.5), cells of the endodermal lineage form a closed intestinal tube and give rise to the regions of the anterior, middle, and posterior intestine.^22^ From E9.5 to E14.5, the intestinal tube becomes a pseudostratified layer and the intestinal epithelium and mesenchyme proliferate rapidly.^23^ Lineage tracing shows that Lgr5^+^ ISC first appears at E12.5.^24^ Around E16.5, epithelial cells differentiate into absorptive and secretory intestinal epithelial cells. By 14 days after birth (P14), the intestinal crypts and villus structure resemble those of the adult mouse.^23^ The fetal intestinal organoids derived from the early fetal intestine, fetal organoids have a greater area for proliferation, evenly dispersed proliferating cells on the surface and present a spherical structure without budding.^25^ The proliferation of adult intestinal organoids is restricted to the crypt structural domains, and the crypt domains extend outward to form branching structures known as budding (Figure 1). Fetal intestinal organoids and adult intestinal organoids display notable differences in their transcriptomes.^25^ But when intestinal epithelial cells sustain injury, adult intestinal organoids undergo transient reprogramming to generate fetal‐like organoids that proliferate rapidly to promote damage repair.^16^, ^25^, ^26^ We have effectively restored the damaged intestine by transplanting and colonizing intestinal organoids into the injured intestinal mucosa.^3^

### 2.2 Characteristics of the intestine during injury

The intestinal epithelium acts as a barrier against the harsh environment. It hosts a diverse and complex community of microorganisms, chemicals, and metabolites, some of which possess pathogenic and toxic properties.^27^ The intestinal tract is very susceptible to injury from various external factors, including mechanical stress, radiation, chemotherapy agents, pathogenic bacteria, and intestinal diseases. Such insults cause damage to the intestinal epithelium.^1^ However, the epithelium possesses a remarkable capacity for rapid self‐repair, efficiently restoring its structural integrity and thereby mitigating the risk of infection.^28^ Intestinal surgery, irritable bowel syndrome, intestinal obstruction, and Crohn's disease (CD) result in elevated intraluminal pressure, leading to deformation of the intestinal mucosa. This deformation can cause mucosal atrophy, which negatively impacts both intestinal physiology and the healing processes.^29^ In mice, mechanical stress resulting from simulated colon tumor expansion activates WNT target genes in adjacent normal tissue. This process accompanied by crypt enlargement during the formation of early tumorous aberrant crypt foci.^30^ Irradiation and 5‐fluorouracil (5‐FU) are widely used anticancer treatments in clinical practice.^31^, ^32^ However, the intestine is particularly sensitive to these treatments. After irradiation, the apoptotic factors such as p53, poly ADP‐ribose polymerase 1 (PARP‐1), and caspase‐3 being highly expressed in crypt cells.^33^ High doses of radiation can lead to the loss of Lgr5^+^ ISCs and the elimination of proliferating progenitor cells, resulting in crypt atrophy.^34^ After irradiation, endothelial cells generate significant amounts of reactive oxygen species (ROS) and secrete ceramide and acidic sphingomyelinase, leading to increased vascular permeability and tissue hypoxia.^35^, ^36^ Immune cells, such as M1 macrophages and mast cells are activated, triggering a long‐term inflammatory response and inhibiting crypt regeneration.^37^, ^38^ Acute inflammation and IBD can also cause the loss of Lgr5^+^ ISCs.^39^ Irradiation can also disrupt the balance of the gut microbiota, leading to an increased abundance of pathogenic bacteria, such as *Helicobacter* and *Clostridium*. While concurrently reducing the relative abundance of beneficial phyla like *Bacteroidetes* and *Firmicutes*.^40^ Bacterial, viral, or parasitic infections can cause extensive damage to the intestinal tract, inducing the formation of wound‐associated epithelial (WAE) cells. These cells rapidly cover the damaged regions, facilitating the swift repair of the intestinal epithelium.^41^, ^42^

However, earlier studies have demonstrated that Lgr5^+^ ISC are not essential for intestinal regeneration. Even when Lgr5^+^ ISC are depleted following radiation‐induced damage, the intestinal epithelium retains the ability to recover.^43^ Studies show that differentiated cells can undergo dedifferentiation upon injury, giving rise to stem cells with self‐renewal capacity.^44^, ^45^, ^46^ Additionally, dormant reserve stem cells located at position +4 are activated after damage to perform stem cell functions.^47^ Current findings suggest isthmus progenitors within the crypts are now considered the primary contributors to intestinal homeostasis and regeneration.^48^ Following ISC loss after injury, differentiated epithelial cells can revert to a stem cell state with self‐renewal capabilities. Secretory lineage cells such as delta‐like canonical Notch ligand 1 (*Dll1*) and atonal basic helix‐loop‐helix (bHLH) transcription factor 1 (*Atoh1*) secretory progenitors, as well as lysozyme 1 (*Lyz1* ^+^) Paneth cells and goblet cells, have the potential to generate fully functional ISCs.^44^, ^45^, ^46^, ^49^ After the depletion of Lgr5^+^ ISCs, cells marked by *Bmi1*, mouse telomerase reverse transcriptase (*mTert*), homeodomain only protein (*Hopx*), or leucine‐rich repeats and immunoglobulin‐like domains 1 (*Lrig1*) are activated at the +4 position above the crypts. These cells take on stem cell functions and contribute to epithelial repair.^13^, ^47^, ^50^, ^51^, ^52^, ^53^, ^54^, ^55^, ^56^ A population of cells highly expressing clusterin (*Clu*) has also been found in the intestinal epithelium after irradiation. These cells are known as the revival stem cell.^26^ After irradiation, Lgr5^+^ ISC ablation mediated by diphtheria toxin (DT) or dextran sulfate sodium salt (DSS) injury, these cells undergo transient expansion, reviving the homeostatic stem cell compartment and replenishing the damaged intestinal epithelium. During the repair process, the damaged epithelial cells undergo transient reprogramming, resulting in the upregulation of fetal and regeneration markers, while the markers of adult stem cell and differentiation are suppressed.^16^, ^26^ When cultured in vitro, parasite‐infected crypts can develop a spheroid with a fetal‐like transcriptome, known as fetal‐like organoid.^57^ The WNT, Notch, and YAP/transcriptional coactivator with PDZ‐binding motif (TAZ) signaling pathways are currently recognized as key contributors to intestinal damage repair.^3^, ^16^, ^58^, ^59^ The WNT pathway is essential for maintaining stem cell homeostasis, and its inhibition impairs crypt repair following irradiation.^58^ Upon intestinal injury, Notch pathway target genes such as Hes family bHLH transcription factor 1 (*Hes1*) and the Notch intracellular domain (NICD) are upregulated.^46^ The YAP/TAZ pathway plays a critical role in damage repair.^60^ YAP expression increases rapidly after irradiation and DSS injury, and blocking YAP/TAZ activity hinders the repair process.^16^ Additionally, inhibiting YAP/TAZ can prevent the overactivation of the WNT pathway during injury and restrain stem cells from excessively differentiating into Paneth cells.^61^ The genes regulated downstream of YAP are markedly enriched in small intestinal organoids derived from embryonic mice. Furthermore, YAP overexpression in adult organoids significantly enhances organoid formation efficiency and induces fetal‐like organoids.^62^, ^63^ However, the mechanisms by which these pathways interact to coordinate tissue repair remain poorly understood.

### 2.3 Characteristics of ISC and the intestine during aging

Aging is accompanied by progressive changes in the intestines, which are linked to a higher incidence of intestinal diseases, including malnutrition, chronic constipation, and colorectal cancer (CRC).^64^, ^65^, ^66^, ^67^ These age‐related alterations in the intestines encompass loss of stem cells, downregulation of cellular metabolism, dysfunction of the intestinal barrier, and dysbiosis of the gut microbiota.^68^ The aging process of the intestine is characterized by notable morphological alterations. In aged mice, the villus are significantly elongated and twisted compared the young.^69^ Additionally, ISCs tend to differentiation of secretory lineage. A decrease in Notch1 expression was observed in the aged intestinal epithelium, which in turn leads to a higher abundance of Paneth cells and goblet cells.^70^ Mechanistically, the excessive accumulation of proinflammatory cells in the lamina propria of the aging intestine leads to elevated levels of interferon gamma (IFNγ). This increased IFNγ activates signal transducer and activator of transcription 1 (*Stat1*) in ISCs, resulting in the overexpression of secretory lineage marker genes and causing ISC to preferentially differentiate into secretory cells.^71^ The number of apoptotic cells increases in senescent crypts, while the number of ISCs decreases.^70^ This is accompanied by a functional decline. Compared to young mice, 17–24‐month‐old mice exhibited a notable 62% reduction in Lgr5‐GFP^High^ ISCs,^72^ accompanied by a decline in the ISC marker, olfactomedin 4 (OLFM4).^69^, ^70^, ^73^ After two rounds of irradiation (10 Gy + 10 Gy), the rate of crypts regeneration is significantly lower in aged mice (18–22 months).^70^ In vitro, crypts isolated from aged mice (24 months) demonstrate a markedly reduced ability to form organoids. Crypts from young mice can produce organoids that can be passed down through successive generations. While organoids from senescent crypts form fewer colonies after three to four passages. That indicates a decline in ISC function with aging.^74^, ^75^, ^76^ Several signaling pathways are involved in regulating the downregulation of senescent ISC function, such as perturbation of WNT, mammalian target of rapamycin (mTOR) signaling, and reduction of fatty acid oxidation (FAO). In senescent ISCs, Paneth cells, and mesenchymal cells, the key Wnts such as *Wnt3* are downregulated.^70^ Meanwhile, the extracellular WNT inhibitor Notum is significantly upregulated in senescent Paneth cells, leading to reduced WNT signaling and a consequent WNT deficiency in aged ISCs.^74^ In aged mice (17.5 months), mechanistic target of rapamycin complex 1 (mTORC1) is hyperactivated in ISCs and progenitor cells. Hyperactivated mTORC1 drives villus aging by inhibiting ISC/progenitor cell proliferation through amplifying the MKK6‐p38‐p53 stress response pathway.^77^ Aged ISCs have impaired FAO and reduced lipid utilization, while FAO agonists and short‐term fasting can activate FAO and restore and improve the activity of ISCs and progenitor cells.^73^ In addition, the N‐terminal domain of protein tyrosine kinase 7 (PTK7) released by senescent fibroblasts is a senescence‐associated secretory phenotype (SASP). PTK7 activates WNT/Ca^2+^ signaling, which in turn triggers nuclear translocation of YAP, reduces stem cell activity and differentiation, and ultimately impairs crypts formation.^78^ It is evident that ISC function declines with age, and is accompanied by a compromised intestinal barrier and immune function.^79^, ^80^, ^81^, ^82^ These declines may be a key factor contributing to the development of age‐related intestinal diseases and possibly even intestinal cancer.

## 3 REGULATION OF INTESTINAL HOMEOSTASIS

The health of the intestinal tract relies on the maintenance of intestinal homeostasis, which is largely supported by the autoregulation of intestinal epithelial cells and closely linked to the regulation of the tissue microenvironment. Recent studies have shown that nutrient metabolism is crucial for stem cell self‐renewal and the preservation of intestinal homeostasis.^83^ Several transcription factors have been identified as key players in gut development and ISC fate.^1^, ^84^, ^85^, ^86^ The intestinal tissue microenvironment consisting of nonepithelial components such as mesenchymal cells, immune cells, and the diverse gut microbiota.^28^ This microenvironment interacts with intestinal epithelial cells to preserve ISC function and enhance regenerative responses. These interactions are governed by various signaling pathways, essential for intestinal repair, particularly in response to injury or disease.^87^, ^88^, ^89^ Recent studies have identified cellular metabolism as a fundamental regulator of ISC homeostasis,^83^ positioning nutrient metabolism as a promising avenue for future research.

### 3.1 Regulation of intestinal homeostasis by epithelium

The maintenance of intestinal homeostasis relies on the self‐regulation on ISCs, which is closely tied to stem cell fate determination. ISCs undergo self‐renewal to produce new ISCs while also differentiating into progenitor cells with proliferative capacity. These progenitor cells continue to differentiate, eventually giving rise to mature epithelial cells.^90^, ^91^ The processes of ISC self‐renewal and differentiation are maintained in a dynamic balance, and any disruption to this balance can compromise intestinal homeostasis.^92^, ^93^ Numerous transcription factors play a critical role in determining ISC fate. In Beumer and Clevers’ review, the key transcription factors and classical signaling pathways that regulate the differentiation of ISCs are described in detail.^1^ Recently, more key genes and transcription factors have been identified as crucial regulators of intestinal development and ISC fate determination. Our laboratory has identified hepatocyte nuclear factor 4 (HNF4) as a key transcription factor that regulates intestinal development and maintains intestinal function.^83^, ^94^, ^95^, ^96^ Embryonic ablation of HNF4 induces extensive cytoskeletal reorganization across multiple organs, including the intestine, kidneys, and yolk sacs, culminating in the absence of brush borders.^95^ Moreover, *Hnf4αγ^DKO^* at E18.5 results in pronounced intestinal dysplasia, characterized by a translucent and distended intestinal lumen, as well as a loss of villus differentiation.^85^ In the intestine of adult mice, the HNF4–SMAD4 signaling pathway plays a crucial role in regulating the fate of enterocytes. The deletion of HNF4 promotes ISC differentiation into progenitor and secretory cell lineages.^96^ Additionally, HNF4 is instrumental in the activation of intestinal FAO and supports ISC self‐renewal.^83^ Similarly, deletion of Krüppel‐like factor 5 (KLF5) results in the loss of ISC identity, while the *Lgr5^ΔKlf5^* causes accelerated ISCs proliferation but a loss of self‐renewal capacity. This imbalance leads to excessive progenitor cell proliferation within the crypts, eventually exhausting ISCs and triggering premature differentiation of enterocyte.^86^ Caudal type homeobox 2 (CDX2) and TATA‐box‐binding protein‐associated factor 4 (TAF4) are both critical transcription factors in establishing the gut. CDX2 is essential for the interplay between intestinal epithelium and mesenchyme, and its ectopic expression in the gastric epithelium can induce a transformation into intestinal epithelium.^97^ When deletion of *Cdx2* during embryonic development, the transcriptome of the mutant ileum is highly similar to the esophagus, with intestinal epithelial cells in the distal small intestine being replaced by keratinocytes.^98^ Similarly, loss of *Taf4* in mice at E18.5 results in a villus‐deficient intestine with a flat epithelium. In adult mice, the ablation of *Taf4* in the epithelium leads to a highly fragile intestine, with the cecum becoming filled with gas.^99^ Dachsous cadherin‐related 1 (*Dchs1*) and FAT atypical cadherin 4 (*Fat4*) are essential for the formation of villus and mesenchymal clusters. In E15.5 mice, *Dchs1^CKO^* and *Fat4^CKO^* led to abnormally flat villus, with mesenchymal cells beneath the villus failing to cluster properly. Instead, these disorganized mesenchymal cells prevent the formation of fully functional villus.^100^ Special AT‐rich sequence‐binding protein 2 (SATB2) and metastasis‐associated 1 family member 2 (MTA2) are crucial transcription factors involved in regulating the fate of colon stem cells and colon formation. In adult mice, deletion of *Satb2* causes colon stem cells to transform into ileum‐like stem cells, resulting in the conversion from colon cells to small intestinal cells.^84^ While the small intestine is responsible for absorbing nutrients such as lipids and carbohydrates, the colon primarily absorbs electrolytes. Loss of *Mta2* in the colon of adult mice leads to the activation of *Hnf4α*, which upregulates the expression of lipid transporter fatty acid‐binding protein 6 (FABP6) and microsomal triglyceride transfer protein (MTTP), consequently increasing lipid uptake in the colon.^101^ Furthermore, the forkhead box A (FOXA) shapes the intestinal microbiota in mice by controlling the glycosylation of intestinal epithelial cells. When *Foxa1* and *Foxa2* are knocked out in intestinal epithelial cells, the glycosylase network is disrupted, leading to drastic changes in microbial composition and spontaneous IBD.^102^

### 3.2 Regulation of intestinal homeostasis by mesenchymal cells

**FIGURE 2:**
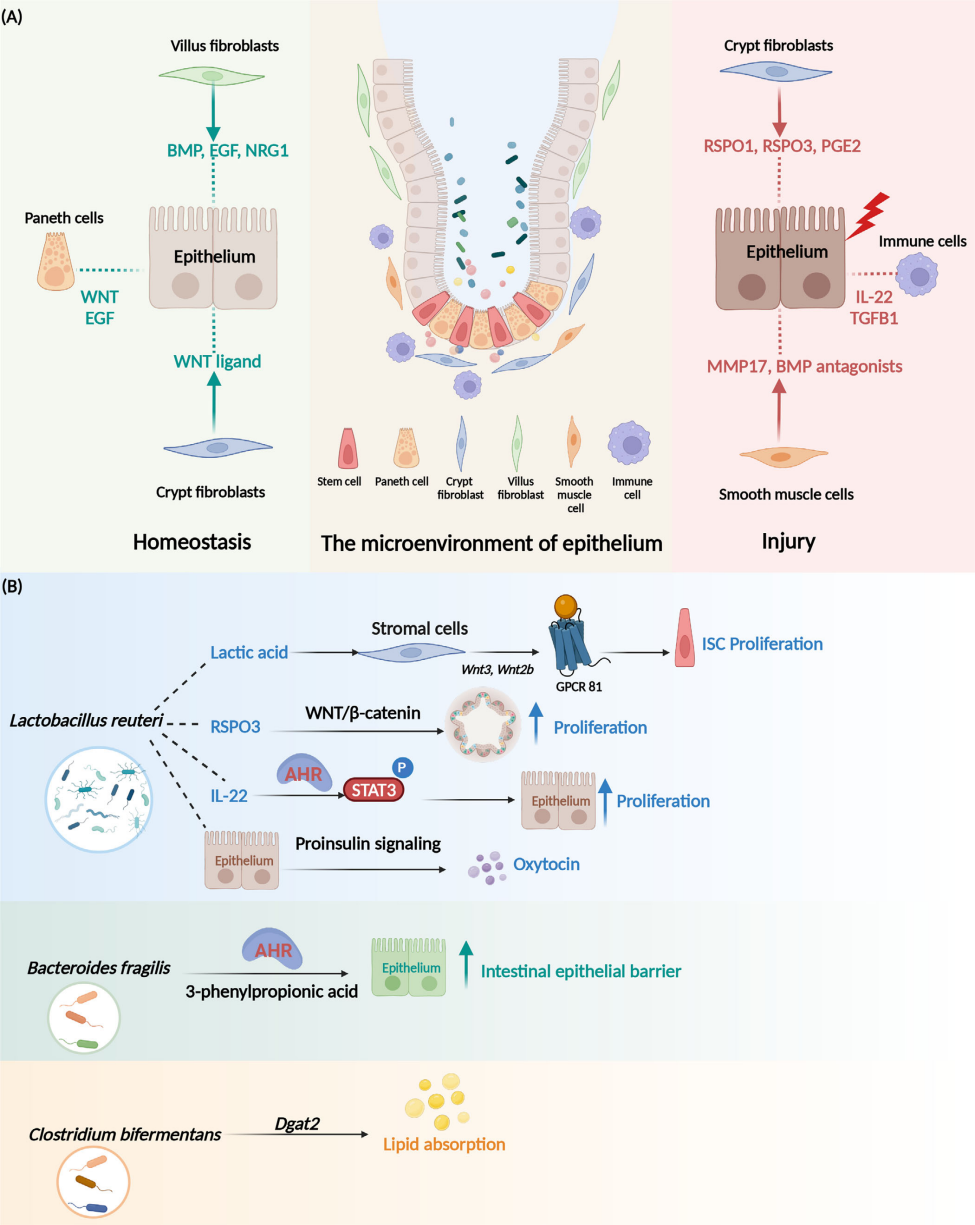
Regulation of intestinal homeostasis by microenvironment. (A) The intestinal microenvironment comprises stromal cells and gut microbiota. Stromal cells include mesenchymal and immune cells. The mesenchymal cells encompassing fibroblasts, myofibroblasts, pericytes, smooth muscle cells, and mesenchymal stem cells. Paneth cells and crypt myofibroblasts secrete wingless‐related integration site (WNT) and epidermal growth factor (EGF) ligands, which are essential for the growth and maintenance of intestinal stem cells (ISCs). Villus fibroblasts contribute to epithelial differentiation by secreting bone morphogenetic protein (BMP) ligands and the EGF family ligand neuregulin 1 (NRG1). Upon intestinal injury, fibroblasts upregulate the secretion of R‐spondin3 to promote tissue repair. MAP3K2‐regulated intestinal stromal cells (MRISC) are located at the base of colonic crypts. These cells enhance WNT signaling through the upregulation of R‐spondin1. *Ptgs2* ^+^ fibroblasts secrete prostaglandin E2, which activates the Yes‐associated protein (YAP) pathway and stimulates epithelial regeneration. After radiation‐induced damage, macrophages accumulate at the injury site, releasing transforming growth factor beta 1 (TGFB1) to facilitate tissue repair. Innate lymphocyte types 3 (ILC3s) activate postinjury and secrete interleukin‐22 (IL‐22) to further support the regenerative process. Smooth muscle cells contribute to intestinal repair by secreting BMP antagonists and matrix metallopeptidase 17 (MMP17), which together enhance YAP signaling. (B) *Lactobacillus reuteri* produces lactic acid, which activates G‐protein‐coupled receptor 81 (GPR81) receptors on stromal cells, leading to the upregulation of *Wnt2* and *Wnt3a* expression. This activation enhances the WNT/β‐catenin signaling pathway, thereby promoting ISC self‐renewal and organoid proliferation. Furthermore, *Lactobacillus reuteri* stimulates lamina propria lymphocytes to secrete IL‐22, which activates the signal transducer and activator of transcription 3 (STAT3) pathway and accelerates proliferation of intestinal epithelial cells. Additionally, *Lactobacillus reuteri* promotes oxytocin secretion through proinsulin signaling mechanisms. *Bacteroides fragilis* produces 3‐phenylpropionic acid derivatives, which contribute to the maintenance of intestinal barrier integrity by activating the aryl hydrocarbon receptor (AhR) signaling pathway. *Clostridium bifermentans* enhances lipid absorption by upregulating diacylglycerol O‐acyltransferase 2 (*Dgat2*) expression, thereby increasing oleic acid uptake and regulating lipid metabolism.

The maintenance of intestinal homeostasis is not only dependent on the regulation of epithelial cells. The tissue microenvironment also plays a crucial role (Table 1). Intestinal mesenchymal cells work synergistically to regulate intestinal homeostasis through interactions with epithelial cells and immune cell.^103^ Mesenchymal cells are a highly heterogeneous population of cell types (Figure 2), including fibroblasts, myofibroblasts, pericytes, smooth muscle cells, and mesenchymal stem cells.^88^ Crypts myofibroblasts located at the base of crypts, and they produce WNT ligands that maintain ISC growth.^104^ Platelet‐derived growth factor receptor alpha (*Pdgfrα* ^+^) mesenchymal cells express high levels of R‐spondin3, which amplifies WNT signaling.^105^ Villus fibroblasts (VF) are a major source of BMP ligands.^106^, ^107^ *Pdgfrα*‐high VF progenitors secrete the epidermal growth factor (EGF) family ligand neuregulin 1 (NRG1), which induces ISC differentiation toward to the secretory cell lineage.^108^ NGR1 was strongly upregulated in macrophages and *Pdgfrα* ^+^ stromal cells during injury, and activation of the mitogen‐activated protein kinase (MAPK) pathway and phosphatidylinositol 3‐kinase (PI3K)/AKT, also known as protein kinase B (PKB) signaling supported proliferation of stem and progenitor cells during repair.^87^ Irradiation and DSS resulted in a large expansion of fibroblasts near the injury site and expression of large amounts of R‐spondin3 to promote intestinal repair.^49^, ^109^ There is a subset of MAP3K2‐regulated intestinal stromal cells (MRISC) in the basal part of colonic crypts that upregulate R‐spondin1 expression through the ROS‐MAP3K2‐Extracellular signal‐regulated kinase 5 (ERK5)‐KLF2 pathway and enhance WNT signaling. It maintains the function of Lgr5^+^ ISC, and resists DSS‐induced colitis.^103^ Smooth muscle cells in the mucosal muscularis propria, located near the base of colonic crypts, serve as a source of BMP antagonists while also enhancing the YAP signaling pathway. These cells express the matrix metallopeptidase 17 (MMP17), which remodels the extracellular matrix and plays a crucial role in tissue repair.^89^ Beneath the crypts lies a rare class of prostaglandin‐endoperoxide synthase 2 (*Ptgs2* ^+^) fibroblasts that secrete prostaglandin E2 (PGE2), which activates YAP and promotes the expansion of the lymphocyte antigen 6 family member S (*Ly6a*) cells population, resembling a reserve stem cell pool.^88^

**TABLE 1:** Regulation of intestinal homeostasis by microenvironment.

Classification	Microenvironment component	Effect on intestinal	Refs.
Mesenchymal cells	*Pdgfrα* ^+^ mesenchymal cells	*Pdgfrα* ^+^ mesenchymal cells express high levels of R‐spondin3, which amplifies WNT signaling.	^105^
	Villus fibroblasts	Villus fibroblasts are the major source of BMP ligands that promote intestinal epithelial cell differentiation.	^106^, ^107^
	*Pdgfrα*‐high villus fibroblasts	*Pdgfrα*‐high villus fibroblasts secrete the EGF family ligand NRG1, which induces ISC differentiation toward to the secretory cell lineage.	^108^
	MRISC	MRISC can upregulate R‐spondin1 expression and enhance WNT signaling.	^103^
	Smooth muscle cells	Smooth muscle cells can secrete BMP antagonists, enhance the YAP signaling pathway and MMP17, remodeling the extracellular matrix.	^89^
	*Ptgs2* ^+^ fibroblasts	*Ptgs2* ^+^ fibroblasts secrete PGE2, which activates YAP.	^88^
	Mesenchymal stem cell	Mesenchymal stem cells secrete CCL2 and CXCL12, which promote the M2 polarization of macrophages.	^110^
Immune cells	Macrophages	Macrophages secrete high levels of TGFB1, and TGFB1 activates the regenerative transcription factors YAP.	^3^
	ILC3	ILC3 secretes IL‐22, which induces STAT3 phosphorylation and promotes ISC proliferation.	^111^
Gut microbiota	*Lactobacillus reuteri*	*Lactobacillus reuteri* stimulates the secretion of IL‐22, induces the expression of R‐spondins, enhances the expression of antimicrobial peptides, and promotes oxytocin secretion.	^112^, ^113^, ^114^
	*Bacteroides thetaiotaomicron*	*Bacteroides thetaiotaomicron* enhances intestinal transit after colonization of GF mice.	^115^
	*Bacteroides fragilis*	*Bacteroides fragilis* derivatives 3‐phenylpropionic acid and promotes the integrity of the intestinal epithelial barrier by activating AhR signaling.	^116^
	*Clostridium bifermentans*	*Clostridium bifermentans* increases the expression of the *Dgat2*, enhances oleic acid uptake and regulates lipid absorption.	^117^

Abbreviations: AhR, aryl hydrocarbon receptor; BMP, bone morphogenetic protein; CCL2, C–C motif chemokine ligand 2; CXCL12, C–X–C motif chemokine ligand 12; EGF, epidermal growth factor; ILC3, innate lymphocyte type 3; IL‐22, interleukin‐22; ISC, intestinal stem cell; MMP17, matrix metallopeptidase 17; MRISC, MAP3K2‐regulated intestinal stromal cells; NRG1, neuregulin 1; PGE2, prostaglandin E2; STAT3, signal transducer and activator of transcription 3; TGFB1, transforming growth factor beta 1; WNT, wingless‐related integration site.

### 3.3 Regulation of intestinal homeostasis by immune cells

During intestinal injury, the immune system activates (Figure 2) and leads to the recruitment of a substantial number of immune cells to the site of damage.^118^, ^119^ Our study found that on day 2 after irradiation, macrophages accumulate at the base of crypts. These cells secrete high levels of transforming growth factor beta 1 (TGFB1) and activates the regenerative transcription factors YAP‐SRY‐Box transcription factor 9 (SOX9) circuit. It induces the epithelium to undergo reprogramming, and generates fetal‐like cells to promote intestinal repair.^3^ Additionally, bone marrow‐derived mesenchymal stem cells secrete C–C motif chemokine ligand 2 (CCL2) and C–X–C motif chemokine ligand 12 (CXCL12), which promote the polarization of M2 macrophages and activate interleukin‐10 (IL‐10^+^) T cells and IL‐10^+^ B cells within the intestinal tract, exerting an inhibitory effect on colitis.^110^ Recent findings highlight the critical role of innate lymphocyte type 3 (ILC3) and interleukin‐22 (IL‐22) in the process of intestinal regeneration. After injury, ILC3 activates and secretes IL‐22. It induces signal transducer and activator of transcription 3 (STAT3) phosphorylation and promotes ISC proliferation.^120^ Additionally, ILC3 detects lysophosphatidylserine (LysoPS) released from apoptotic neutrophils through its membrane receptor G‐protein‐coupled receptor 34 (GPR34), which triggers the PI3K‐AKT and RAS‐ERK pathways, leading to the release of IL‐22 and initiation of tissue repair.^111^ Furthermore, both adrenalines secreted by intestinal nerve cells and viral infections can activate ILC3, drive IL‐22 production and enhance the regeneration of intestinal epithelium.^121^, ^122^

### 3.4 Regulation of intestinal homeostasis by gut microbiota

The intestinal lumen harbors a vast and diverse community of gut microorganisms, which play a crucial role in maintaining gut health and function.^123^, ^124^ These gut microbiotas are essential for the establishment of a mature immune system during early embryonic development.^125^, ^126^ The gut microbiota stimulates the formation of the intestinal capillary network.^127^ Chorionic capillaries in germ‐free (GF) mice are underdeveloped after weaning and remain so into adulthood.^127^ Additionally, early gut microbiota induces the secretion of antimicrobial peptides by Paneth cells, shaping the innate immune response in the intestinal epithelium.^128^ Microbes during the early stages of intestinal development also activate the WNT pathway, enhance the expansion of Lgr5^+^ ISC. They promote the growth of intestinal organoids and facilitate regeneration of crypts after injury.^129^ The intricate interactions between specific gut microbiota an ISC niches have been increasingly recognized as essential for maintaining intestinal homeostasis.^130^, ^131^ Two excellent reviews offer comprehensive insights into the intricate crosstalk between ISCs and the gut microbiota, elucidating the specific molecular mechanisms involved in this dynamic interaction.^130^, ^131^ Here, we have added the latest discoveries about the gut microbiota (Figure 2). The intestinal probiotic *Lactobacillus reuteri* has been shown to stimulate the secretion of IL‐22 from lamina propria lymphocytes (LPLs), leading to the activation of STAT3 and subsequent acceleration of intestinal epithelial cell proliferation. Additionally, *Lactobacillus reuteri* promotes the proliferation of intestinal organoids by inducing the expression of R‐spondins under homeostatic conditions, thereby sustaining the activation of the WNT/β‐catenin pathway.^112^ This probiotic also reduces the secretion of proinflammatory cytokines in the intestine, lowers concentrations of serum lipopolysaccharides (LPS), enhances the expression of antimicrobial peptides, and inhibits the colonization of *Citrobacter rodentium*.^112^ Furthermore, a recent study revealed that *Lactobacillus reuteri* can promote oxytocin secretion from human intestinal tissues and organoids via proinsulin signaling.^113^ Lactic acid produced by *Bifidobacterium* and *Lactobacillus* has been shown to stimulate Paneth and stromal cells via G‐protein‐coupled receptor 81 (GPR81), thereby promoting epithelial regeneration. Furthermore, preadministration of lactic acid has demonstrated protective effects against irradiation‐induced damage.^114^ While *Bacteroides thetaiotaomicron* produces tryptamine, which enhances intestinal transit after colonization of GF mice.^115^ *Bacteroides fragilis*—a species known to reinforce the intestinal epithelial barrier. It can derivative 3‐phenylpropionic acid and promotes the integrity of the intestinal epithelial barrier by activating aryl hydrocarbon receptor (AhR) signaling.^116^ Additionally, various specialized gut microbiota is implicated in host metabolism. The *Clostridium bifermentans* and its metabolites increase the expression of the diacylglycerol O‐acyltransferase 2 (*Dgat2*), which is associated with lipid absorption in the small intestine. It can enhance oleic acid uptake and regulate lipid absorption.^117^ In GF mice, the absence of intestinal microbiota impairs glucose uptake and storage capacity in skeletal muscle. It leads to an increase in brown adipogenesis, which prevents obesity. However, this metabolic alteration is accompanied by reduced ATP production, resulting in diminished exercise capacity. The specific microbial strains responsible for regulating this process remain unidentified.^132^

## 4 METABOLIC REGULATION OF INTESTINAL HOMEOSTASIS

The process of renewing small intestine epithelial cells is characterized by high dynamism, and this constant turnover is closely linked to intense energy metabolism. The metabolism of carbohydrates, lipids, and proteins serves as a vital source of energy for the body. In addition, the metabolic products participate as precursors in the production of substances for regulating the body's physiological function. We previously conducted a study that discovered that HNF4, a transcription factor responsible for regulating the process of FAO, enhances the renewal of ISCs. This finding suggests that alterations in nutrition and metabolic pathways have the potential to impact the quantity and renewal capacity of ISCs.^83^ The core of intestinal homeostasis regulation depends on ISCs, which are highly sensitive to the nutritional status of the organism. Therefore, it is critical to understand how the nutritional pathways (Table 2) and their metabolites affect the ISCs, as well as intestinal epithelial cells.

**TABLE 2:** Effects of key metabolism‐related proteins on intestinal stem cell (ISC) self‐renewal and intestinal homeostasis.

Metabolic pathway	Protein	Metabolism function	Phenotype of genetic model	Refs.
Mitochondria and oxidative phosphorylation	YY1	A transcription factor that regulates mitochondrial structure and OXPHOS	The intestinal epithelium deficiency decreases ISC number and villus length.	^133^, ^134^
	FOXO	A transcription factor that regulates mitochondria activity and division	The intestinal organoids deficiency decreases ISC markers and increases secretory cell markers.	^135^
	HSP60	A mitochondrial chaperone	The intestinal epithelium deficiency in mice causes transient loss of ISCs and initiates a transition of ISCs toward a Paneth cell‐like phenotype.	^136^, ^137^
	DARS2	An aspartyl‐tRNA synthetase that involves in mitochondria respiratory chain assembly	Intestinal epithelium‐specific deletion results in accumulation of lipid droplets in the proximal small intestine and severe intestinal lesions with reduced numbers of stem, proliferative, secretory, and absorptive cells.	^138^
	UCP4C	Uncoupling protein	Overexpression in the *Drosophila* intestine extends lifespan and rescues the intestinal aging phenotype.	^139^
Glucose metabolism	HK2	Generation of glucose 6‐phosphate (G6P)	Intestinal epithelium‐specific deletion *Hk2* leads to a decrease in crypt organoid‐forming capacity, ISC conversion to secretory cells.	^140^
	MPC1	Mitochondrial pyruvate carrier	Specific deletion of *Mpc1* in Lgr5^+^ ISCs expands the intestinal stem cells compartment and increases proliferation.	^141^
Fatty acid oxidation	PRDM16	A transcription factor that drives oxidative metabolism in brown fat	*Prdm16* deletion in mice triggers progenitor cells apoptosis, leading to diminished epithelial differentiation.	^142^
	HNF4	A transcription factor that regulates fatty acid oxidation	Loss of *Hnf4* in the intestinal epithelium triggers Lgr5^+^ stem cell loss, and *Hnf4* DKO organoid lacks branched crypt domains.	^83^
	CPT1A	The carrier of long‐chain fatty acids and a rate‐limiting enzyme for fatty acid oxidation	Long‐term *Cpt1a* deletion decreases ISC numbers and function and impair organoid‐forming capacity.	^73^
	HMGCS2	A rate‐limiting enzyme for ketone bodies synthesis	Loss of *Hmgcs2* in the intestinal epithelium compromises ISC stemness and regeneration after radiation.	^143^
Fatty acid synthesis	FASN	An insulin‐responsive enzyme essential for de novo lipogenesis	Loss of *Fasn* in the colonic epithelium blocks MUC2 production and induces intestinal inflammation.	^144^
	ACC1	An insulin‐responsive enzyme essential for de novo lipogenesis	Loss of *Acc1* in the intestinal epithelium results in impaired crypt structures in the distal intestine (colon and ileum), a decrease in OLFM4‐positive cells, and an increase in the number of Paneth cells.	^145^
Amino acid metabolism	TSC2	A negative regulator of mTORC1	Ablation of *Tsc2* enhances proliferative activity of IECs in intestinal crypts but increases ectopic Paneth cells.	^146^
	TSC1	A negative regulator of mTORC1	Ablation of *Tsc1* of young mice causes premature senescence of intestine.	^77^
	SLC7A5	Reverse transporter of Gln	Intestinal epithelial deletion of *Slc7a5* significantly delays tumor proliferation.	^147^
	SLC25A22	Mitochondrial glutamate transporter	*SLC25A22* promotes proliferation and migration of CRC cells with mutations KRAS.	^148^
	SLC1A3	Asp/Glu transporter	Deletion of *SLC1A3* inhibits human colon cancer cell growth.	^149^

Abbreviations: ACC1, acetyl‐CoA‐carboxylase 1; CPT1A, carnitine palmitoyl‐transferase1 alpha; CRC, colorectal cancer; DARS2, aspartyl‐tRNA synthetase2; FASN, fatty acid synthase; FOXO, forkhead box O; HK2, hexokinase 2; HMGCS2, HMG‐CoA synthase 2; HNF4, hepatocyte nuclear factor 4; HSP60, heat shock protein; MPC1, mitochondrial pyruvate carrier 1; mTORC1, mechanistic target of rapamycin complex 1; OLFM4, olfactomedin 4; PRDM16, PR/SET domain 16; SLC1A3, solute carrier family 1 member 3; SLC25A22, solute carrier family 25 member 22; SLC7A5, solute carrier family 7 member 5; TSC1, TSC complex subunit 1; TSC2, TSC complex subunit 2; UCP4C, uncoupling protein 4C; YY1, neuronal Yin Yang1.

### 4.1 Regulation of ISC by nutrient metabolism

#### 4.1.1 Dietary regulation of ISC self‐renewal

Diet and metabolism intricately intertwine, and the ISCs constantly adjusting its fate decisions based on diet and nutritional status. Various dietary regimens alter the composition of gut microbiota, which in turn impacts the self‐renewal and differentiation of ISCs. This dynamic interaction underscores the profound influence of diet and gut microbiota on ISCs behavior.^150^ Fasting and caloric restriction (CR) have been shown to enhance ISCs self‐renewal and regeneration of small intestinal epithelium.^73^, ^151^ Fasting increased the number of *Lactobacillus* and *Bifidobacterium* in the gut.^152^, ^153^ Oral administration of probiotics (containing *Bifidobacterium* and *Lactobacillus*) induced more ISCs and was accompanied by an increase in the number of Paneth cells and goblet cells. Additionally, *Lactobacillus* protects intestinal epithelium from damage after radiation.^114^ Dietary restriction, such as CR, intermittent fasting, and ketogenic diets, are the most effective antiaging interventions.^154^ CR prevents or reduces the accumulation of senescent cells in the mouse colon and delays kidney aging.^155^, ^156^ The mechanism by which dietary restriction extends lifespan begins with promotion of lipid utilization. Fasting activates peroxisome proliferator‐activated receptors (PPARs), increases FAO in intestinal stem and progenitor cells, and enhances ISC self‐renewal.^73^ In addition, researchers have found that CR leads to an increase in the number of functional Lgr5^+^ ISC that compete for ecological niches.^157^

Ketogenic diet and high‐fat diets (HFD) also promote self‐renewal of ISCs.^143^, ^158^ It was found that beta‐hydroxybutyrate (β‐OHB) produced by the ketogenic diet reinforces Notch signaling, instructing ISC self‐renewal and lineage decisions.^143^ It also inhibits the growth of intestinal *Bifidobacteria*, which leads to a decrease in the level of intestinal CD4^+^ T helper cells 17 (Th17) cells and modulates the intestinal immune response.^159^ HFD increased numbers of ISCs through PPAR‐δ‐dependent activation, and promoted crypt regeneration after irradiation.^158^ However, long‐term HFD activated the growth‐promoting pathways, MAPK/ERK and PI3K/AKT/mTOR, which induced tumor growth in a colon cancer model.^160^ In addition, PPAR‐δ activation induced progenitor cells to take on the characteristics of ISCs, resulting in greater susceptibility to tumorigenesis.^158^ High‐sugar diet increased tumor incidence, and the administration of a high‐fructose beverage to *Apc*‐Min mice significantly increased tumor size.^161^ High‐glucose diet also inhibited self‐renewal of ISCs. Mice provided with glucose‐containing drinking water for 4 weeks exhibited impaired crypt regeneration.^143^

#### 4.1.2 Mitochondria and oxidative phosphorylation

Mitochondria play a crucial role in intracellular oxidative phosphorylation (OXPHOS) for ATP production. Recent studies have revealed that mitochondria also contribute to the regulation of organismal homeostasis by participating in pathways associated with apoptosis and tissue senescence. A study demonstrated that the pyruvate/lactate ratio was higher in ISCs than their neighbors, Paneth cells. This ratio indicates the relative contribution to cellular bioenergetics of mitochondrial respiration versus glycolysis. It suggests that ISCs are more dependent on mitochondrial metabolism as a source of energy.^162^ Intestinal epithelial development is directly influenced by mitochondria, and YY1 is a crucial transcription factor that regulates both the structure and function of mitochondria as well as OXPHOS. Specific deletion of *Yy1* in the epithelium of embryonic mice resulted in severe hypoplasia of the villus and defective differentiation of enterocytes. Additionally, mitochondrial inhibitors hindered the elongation of the villus. YY1 loss in the intestinal epithelium of adult mice resulted in impaired ISCs renewal and led to an imbalance between crypts and villus.^133^, ^134^ The FOXO, a transcription factor, plays a significant role in governing mitochondrial activity and division. Deletion of FOXO inhibits Notch and prompts the transformation of ISC into secretory cells, leading to a reduction in the expression of stem cell markers. This observation demonstrates the intricate regulatory mechanisms by which mitochondria influence ISC fate determination.^135^ Additionally, mitochondrial dysfunction impairs intestinal homeostasis and contributes to intestinal inflammation. DARS2 is involved in mitochondrial respiratory chain assembly. Specific ablation of *Dars2* in the intestinal epithelial cells led to severe mitochondrial impairment.^138^ DARS2 deficiency obstructs the outward transport of lipids absorbed in the small intestine, causing the accumulation of numerous lipid droplets in the proximal small intestine. The *Dars2^KO^* mice manifest severe lesions in the intestine, with a notable decrease in the populations of ISCs, progenitor cells, secretory cells, and absorptive cells.^138^ Loss of the mitochondrial chaperone HSP60 in the intestinal epithelium leads to a decrease in the number of OLFM4‐positive cells, a conversion of Lgr5^+^ ISCs into abnormal Paneth cells,^136^ as well as an induction of macrophage aggregation at the base of the crypts, exacerbating intestinal inflammation.^136^, ^137^

Aging is associated with significant metabolic alteration, and mitochondria playing a central role in age‐related pathologies. Aging is closely linked to mitochondrial dysfunction and mutations in mitochondrial DNA (mtDNA).^163^, ^164^ The increased production of ROS during aging further exacerbates mitochondrial damage, and mitochondria‐derived ROS are thought to be key drivers of cellular senescence.^165^, ^166^ Mitochondrial dysfunction also results in a reduced nicotinamide adenine dinucleotide (NAD^+^/NADH) ratio, leading to the repression of glyceraldehyde 3‐phosphate dehydrogenase (GAPDH), a crucial gene for glycolysis. This repression causes ATP depletion, activation of AMP‐activated protein kinase (AMPK), and subsequent cell cycle arrest.^167^ Factors associated with mitochondrial dysfunction, such as mutations in mtDNA, deletion of mitochondrial chaperone proteins, and mitochondrial protein deacetylases, contribute to cellular senescence. Mice with high mtDNA mutations lead to NAD^+^ depletion in the intestine, and the mice show marked senescence features in the intestine at 8 months. Mechanistically the accumulation of mtDNA depletes NAD^+^, leading to the accumulation of large amounts of unfolded proteins in the mitochondria, thereby exacerbating premature senescence of the small intestine.^72^ The uncoupling protein UCP4C has been identified as a significant factor in extending the lifespan of *Drosophila*. UCP4C maintains stable proliferation of ISCs via decreasing ROS levels.^139^

#### 4.1.3 Glucose metabolism

**FIGURE 3:**
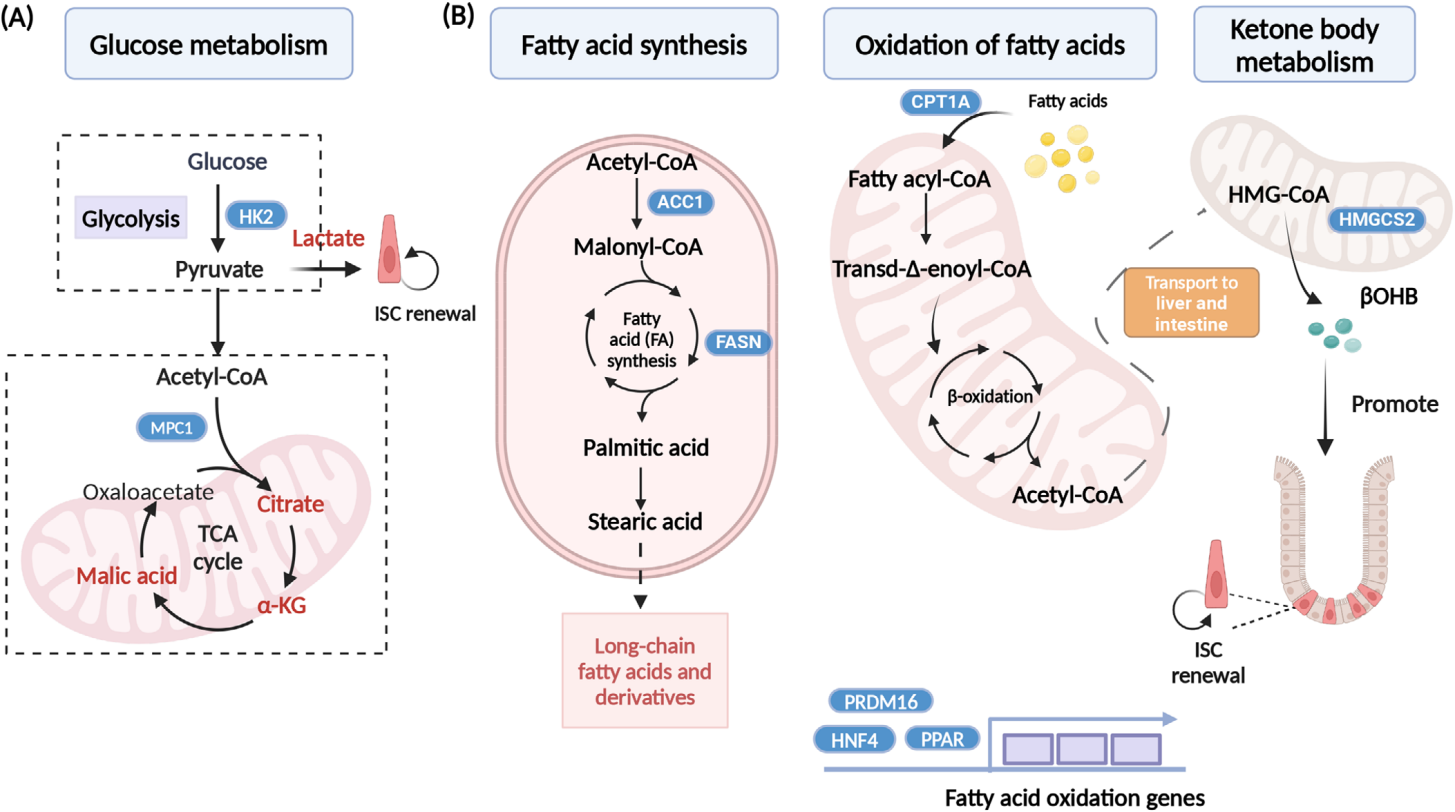
Regulation of intestinal stem cells (ISCs) by glucose, fatty acid, and ketone body metabolism. (A) Regulation of the small intestine by key genes and metabolites involved in glucose metabolism. Paneth cells produce lactate through anaerobic glycolysis to fuel the ISCs and promote ISC self‐renewal. (B) Acetyl coenzyme A carboxylase (ACC) and fatty acid synthase (FASN) are essential enzymes in the pathway of fatty acid synthesis, responsible for producing long‐chain fats and their derivatives. Carnitine palmitoyl‐transferase1 alpha (CPT1A) is involved in the transport of fatty acids into mitochondria. A portion of the acetyl‐CoA produced from fatty acid oxidation is redirected to the liver, where it contributes to ketone body metabolism. In the presence of HMG‐CoA synthase 2 (HMGCS2), beta‐hydroxybutyrate (β‐OHB) is generated to promote ISC self‐renewal. HNF4, hepatocyte nuclear factor 4; PPAR, peroxisome proliferator‐activated receptor; PRDM16, PR/SET domain 16.

The glycolytic metabolism is the primary source of energy for organismal activity, and its intermediates serve as ample precursors for the synthesis of amino acids and nucleotides. While most cancer cells adhere to the “Warburg” metabolism, ISCs predominantly rely on aerobic mitochondrial metabolism, owing to their higher mitochondrial activity. The Paneth cells utilize anaerobic glycolysis to produce large amounts of lactate to fuel ISCs.^162^ Organoid formation was impaired with treatment of sodium dichloroacetate (DCA; glycolysis inhibitor) or OXPHOS inhibitor.^162^ Glycolysis is also closely linked to ISC fate decisions (Figure 3). In the intestinal epithelium, deletion HK2, an enzyme that controls the speed of glycolysis, resulted in reduced ISC self‐renewal and increased secretory cells. Mechanistically, HK2 regulates secretory cell differentiation through increased ATOH1 expression and activation of p38 MAPK.^140^ Interestingly, lactate was able to rescue the phenotype caused by HK2 deletion.^140^ Under aerobic conditions, pyruvate produced from glycolysis is transported into the mitochondria through the mitochondrial pyruvate carrier 1 (MPC1), where it enters the tricarboxylic acid (TCA) cycle and undergoes aerobic catabolism. MPC1 is lowly expressed in the Lgr5^+^ ISC, but its expression significantly rises in the villus as ISC differentiation progresses. Deletion of MPC1 in the Lgr5^+^ ISC leads to an increase in the number of ISCs, but the number of mature differentiated cells (like goblet cells) remains unchanged. MPC1‐deficient organoids derived from *Lgr5*‐EGFP mice maintain a larger proportion of GFP‐positive stem cells,^141^ suggesting that inhibition of MPC1‐mediated pyruvate metabolism drives ISC self‐renewal. But histone acetylation is reduced, and pyruvate metabolism is increased after MPC1 deletion. However, the mechanism by which MPC regulates ISC has not been determined.^141^

#### 4.1.4 Fatty acid metabolism

FAO (Figure 3) is essential for maintaining stem cell function.^168^, ^169^ Our previous study has demonstrated a higher rate of fatty acid uptake in crypts compared to villus. We found deletion of HNF4 in the intestinal epithelium, which plays a critical role in regulating FAO. After loss of HNF4, FAO‐related genes downgraded and resulted in the loss of ISCs and a failure of organoid growth.^83^ The PR/SET domain 16 (PRDM16) along with PPAR proteins regulates small intestinal epithelial renewal via promoting FAO. Intestinal ablation of PRDM16 led to the apoptosis of progenitor cells, significant shortening of the villus, notable lesions in the small intestine, and dysplasia in organoids.^142^ Fasting and CR induce accelerated FAO and activate ketone body metabolism. Mice under calorie restriction exhibit downregulated mTOR pathway activity, which fosters self‐renewal of Lgr5^+^ ISCs at the expense of differentiation and enhance the number of crypts after injury.^73^, ^151^ Twenty‐four‐hour fastening enhances FAO and accelerates production of passaged organoids, but acute ablation of *Cpt1α* inhibited fasting‐induced organoid formation. In homeostatic, acute *Cpt1α* loss is largely dispensable for Lgr5^+^ ISCs maintenance, but chronic deficiency reduces the number and function of ISCs, suggesting the maintenance of ISC function is dependent on long‐term FAO.^73^ HMG‐CoA synthase 2 (HMGCS2) is a rate‐limiting enzyme in ketone body metabolism, and deletion of *Hmgcs2* in the intestinal epithelial cells reduces the number of OLFM4‐positive cells in the crypts and leads to premature conversion of ISCs to secretory cells.^159^ Mechanistically, *Hmgcs2* loss depletes β‐OHB levels in Lgr5^+^ ISCs, leading to Notch inhibition, impairing ISC stemness and biasing them toward secretory lineage differentiation (Figure 3). Exogenous fatty acid supplementation, such as acetate, can rescue impaired organoid caused by defects in FAO‐related genes.^83^, ^142^

Genes involved in fatty acid synthesis have also been shown to regulate ISCs function. Acetyl coenzyme A carboxylase (ACC) is a rate‐limiting enzyme for fatty acid synthesis, and specific deletion of *Acc1* in the intestinal epithelium results in impaired crypt structures in the distal intestine (colon and ileum), a decrease in OLFM4‐positive cells, and an increase in the number of Paneth cells. The organoids deficient in *Acc1* resulted in extensive cell death, and supplementation with acetate failed to rescue this defect. Mechanistically, ACC‐mediated de novo fatty acid synthesis maintains PPAR‐δ/β‐catenin levels in the ISCs for supporting organoid formation and differentiation.^145^ In conclusion, both fatty acid catabolism and synthesis play key roles in maintaining ISC function, and FAO is a key pathway for maintaining ISC function. Deficiency of fatty acid synthase (FASN) in the colonic epithelium blocks mucin 2 (MUC2) production, disrupts the intestinal barrier, and induces enterocolitis.^144^ However, deletion of the oleic acid rate‐limiting enzyme stearoyl‐CoA desaturase (SCD1) in intestinal epithelial cells did not affect ISC self‐renewal and differentiation, but more and larger tumors were found in *Apc*‐Min mice when loss of SCD1.^170^

Overall, lipid metabolism is essential for the maintenance of ISC function. PPAR‐FAO is currently thought to be closely related to stem cell self‐renewal, while pyruvate metabolism and glycolysis promote ISC differentiation into the secretory lineage.^73^, ^135^, ^141^, ^158^ Both short‐term fasting and HFD can increase FAO gene expression and activate PPAR, which in turn increases WNT/β‐catenin expression and activates ISC self‐renewal.^73^, ^158^ HNF4 and PRDM16 can also activate FAO genes and are required for ISC renewal.^83^, ^142^ Inhibition of MPC1, a key gene that inhibits pyruvate metabolism, also promotes FAO and ISC self‐renewal.^141^ OXPHOS is required for the differentiation of intestinal epithelial cells during crypt formation. A decrease in mitochondrial function is associated with an increase in the number of secretory cells.^135^

Recent research has demonstrated that senescence significantly impacts intracellular lipid metabolism. In senescent cells, there is an upregulation of FASN, a key enzyme in fatty acid synthesis, along with related genes involved in β‐oxidation, which may contribute to the increased secretion of the SASP.^171^ Moreover, senescent cells exhibit increased production of polyunsaturated fatty acid (PUFA) derivatives, including oxylipins, dihomo‐prostaglandins, and leukotrienes. These PUFAs activate the renin–angiotensin system (RAS) and promote both SASP secretion and cellular senescence.^172^ It has also been hypothesized that senescent cells utilize PUFA desaturation as a mechanism to convert NADH to NAD^+^, thereby maintaining NAD^+^ redox homeostasis.^173^ Additionally, a recent study revealed an increase in phosphatidyl di(monoacylglycerol) species containing PUFAs across various organs in senescent organisms, while levels of phospholipids containing saturated and monounsaturated fatty acids decrease.^174^ This alteration in lipid composition further underscores the complex metabolic reprogramming that occurs during cellular senescence.

#### 4.1.5 Amino acid metabolism

Amino acids serve as the basic structural units of proteins, and the breakdown products can enter the TCA cycle to provide energy for the organism. Amino acids are also involved in the mTOR signaling pathway, a key pathway for maintaining cell growth, metabolism, and immunity.^175^ The mTOR pathway is actively involved in the renewal of ISCs and the maintenance of intestinal homeostasis.^146^ Glutamine (Gln), arginine (Arg), and leucine(Leu) are known to activate the mTOR pathway.^175^ Disruption of mTOR signaling in the intestinal epithelium resulted in flattened villus and developmental defects in the ileum.^146^ Overactivation of the mTOR pathway by depleting *Tsc1* in the IECs resulted in the development of senescent features of the intestinal tract in young mice, including reduced crypt height and number, reduced villus length and density, and impaired crypt regeneration after irradiation.^77^ Loss of *Tsc2* in the colonic epithelium suppressed WNT pathway, leading to a decrease in the number of Lgr5^+^ colonic stem cells and an increased susceptibility to DSS‐induced colitis. After small bowel resection, the remaining bowel undergoes compensatory effects that results in deeper crypts in the remaining bowel and more cell regeneration. *Tsc1*‐induced overactivation of mTOR causes larger hyperproliferative crypts and leads to uneven distribution of Paneth cells after small bowel resection.^176^ Therefore, mTOR may not be essential for stimulating crypt proliferation under homeostatic conditions, but activation of the mTOR pathway is necessary to repair intestinal damage during injury after irradiation, DSS, or small bowel resection.^176^

Amino acids can produce alpha‐ketoglutarate (α‐KG), which is involved in OXPHOS as well as sugar and lipid synthesis. It is increasingly recognized that tumor growth heavily relies on the uptake of exogenous amino acids, particularly Gln, aspartate (Asp), and serine (Ser). Studies are now delving into amino acid uptake preferences of various tumors, aiming to devise therapeutic strategies that target amino acid metabolism for combating tumors.^175^ Gln is the most consumed nonessential amino acid in the human body. Major consumers of Gln include intestinal cells, immune cells, and tumor cells.^177^ Gln plays an important role in colon cancer growth and invasion. Hypoxia induces high expression of glutaminase 1 (GLS1), the hydrolysis enzyme of Gln, in colon cancer cells, which promotes colon cancer cell migration and tumor growth.^177^

Exogenous Gln deprivation stimulates p53 activation and promotes the expression of the Asp/Gln transporter protein SLC1A3. The absence of SLC1A3 resulted in the inability of cancer cells to utilize Gln, thereby inhibiting colon cancer cell growth.^149^ Gln reverse transporter protein, SLC7A5, maintains intracellular amino acid levels. In *KRAS*‐mutant CRC mice, SLC7A5 functions through transcriptional and metabolic reprogramming to support amino acid requirements for cell proliferation. The intestinal epithelial deletion of *Slc7a5* significantly reduces the number of tumors.^147^ These studies suggest that Gln metabolism and its transporter proteins are attractive targets for the treatment of CRC. Mechanistically, Asp inhibits AMPK‐mediated p53 activation via serine/threonine kinase 11 (STK11) and promotes tumor cell proliferation.^178^ SLC25A22 is an amino acid transport protein that promotes Asp synthesis. Knocking down *SLC25A22* leads to a reduction in Asp biosynthesis and cell proliferation, and an increase in apoptosis, which in turn reduce tumor formation and metastasis in *KRAS*‐mutant CRC cells in mice.^148^ Ser is a precursor for the synthesis of nucleic acids, proteins, lipids, and antioxidants. It is essential for the metabolic processes that support cell growth and survival in cancer development. Restricting Ser effectively induced the production of deoxysphingolipids, leading to a reduction in tumor growth.^179^ In summary, tumor cells favor specific amino acids, suggesting potential treatment avenues by targeting these preferences. Either by knocking down their transport carriers or by restricting their intake could provide a novel approach for cancer therapy. Intriguingly, ISCs have metabolic characteristics similar to those of tumor cells, yet the direct role of these amino acids in regulating ISCs is poorly understood. This raises the pivotal question: Could the convergence of ISCs and tumor cells metabolic pathways hold the key to innovative cancer therapies, reshaping the future of oncology?

### 4.2 Metabolites regulate ISC function

Metabolites are intricately linked to the renewal of ISCs. As mentioned previously, Paneth cells produce large amounts of lactate, which enhances ISC OXPHOS to support stem cell function.^162^ Lactate stimulated the growth of intestinal organoids, and prompted a notable upregulation of *Wnt3* and *Wnt2b* in stromal cells.^114^ Thus, coculture of stromal cells and lactate with intestinal organoids induced larger spherical organoids.^114^ Fatty acids play a critical role in maintaining ISCs, with exposure to palmitic or oleic acid resulting in increased Lgr5^+^ ISCs in the organoids. Palmitic and oleic acid also enhanced generation of organoids. Mechanistically, fatty acids activate PPAR‐δ, which drives the organoid self‐renewal process.^158^ The β‐OHB is key product of ketone body metabolism, supplementation of exogenous β‐OHB increased Lgr5^+^ ISCs and improved the integrity and survival of crypts after injury.^143^ Exogenous supplementation β‐OHB has been shown to upregulate *Oct4* and lamin B1 in vascular smooth muscle and endothelial cell, thereby mitigating aortic senescence in mice.^180^

Amino acids serve not only as building blocks for proteins but also as precursors for the synthesis of a variety of signaling molecules and hormones. Gln is the most abundant nonessential amino acid in the body. In early weaned mice, the self‐renewal process of ISCs was impaired, but exogenous Gln activated the WNT signaling pathway and promoted ISC self‐renewal.^181^ Short‐term Gln deprivation led to a reduction in the crypt domains of the organoids, while long‐term Gln deprivation resulted in organoid atrophy.^181^ Gln also activated the YAP pathway, significantly attenuated intestinal damage in burned mice, and promoted crypt repair and regeneration.^182^ Cysteine (Cys), an essential amino acid, and its derivative—taurine are strongly associated with the aging process. The number of ISCs recovered after taurine supplementation in aging mice.^183^ Methionine (Met) is one of the essential amino acids, and its metabolite, S‐adenosylmethionine (SAM), is critical for ISCs. Met suppressed stem cell proliferation and promoted intestinal organoid differentiation. Additionally, deprivation of Met caused a reduced number of ISCs in *Drosophila*.^139^, ^184^ Tryptophan (Trp) is one of the essential amino acids and a precursor of hormones and neurotransmitters. Serotonin (5‐HT) is derived from Trp, it serves as a neurotransmitter produced and secreted by enterochromaffin cells in the intestine. 5‐HT plays a crucial role in driving self‐renewal of ISCs. Valeric acid, a metabolite produced by gut microbes, stimulates the production of 5‐HT in the intestine. Inhibition of 5‐HT production led to reduced numbers of ISCs, as well as atrophy of crypt and villus. Additionally, it attenuated crypt regeneration after irradiation. Refeeding 5‐HT significantly restored the number of ISCs. Mechanistically, 5‐HT activated macrophages to produce PGE2, which in turn promoted ISC self‐renewal via WNT/β‐catenin.^185^

### 4.3 Metabolic intermediates against pathological stimulation in the intestine

Metabolic intermediates play an important regulatory role in maintaining intestinal homeostasis against pathological stimulation. For example, lactate and pyruvate contribute to increasing intestinal resistance to *Salmonella* infection, potentially enhancing intestinal immunity.^186^ The α‐KG and malate, key products of the TCA cycle, have recently been shown to both activate macrophages and promote damage repair in the intestinal mucosa.^187^ Supplementation with α‐KG leads to a reduction in the abundance of pathogenic bacteria, including *Ehrlichia* and *Enterococcus*. It also enhances intestinal barrier function and facilitates the restoration of colon damage induced by DSS.^188^ Gln also plays an important role in maintaining the intestinal barrier during intestinal injury. Gln supplementation increases blood glutathione (GSH) levels, reduces intestinal permeability, and restores small intestinal barrier function after ischemia/reperfusion injury.^189^ Deprivation of Gln or inhibition of Gln synthetase in Caco‐2 cells significantly diminishes the expression of tight junction proteins.^190^ However, the specific mechanism by which Gln regulates intestinal barrier function is poorly understood. Additionally, the abundance of the Trp metabolites, xanthurenic acid (XANA) and kynurenic acid (KYNA), is negatively correlated with IBD. Supplementation with XANA and KYNA increases mitochondrial respiration, promotes intestinal epithelial cell proliferation, and facilitates repair in a DSS‐induced colitis model. XANA and KYNA also facilitate the differentiation of Th17 cells, which strengthens the tight junctions of the intestinal epithelium to maintain the intestinal barrier.^191^

Metabolites also play roles in colorectal tumors. For example, α‐KG promotes DNA and histone H3K4me3 hypomethylation, inhibits WNT signaling, and promotes cellular differentiation. Moreover, it inhibits CRC tumor growth and shows promising anticancer effects.^192^ Oleic acid reduces tumors in *APC*‐Min mice,^170^ while arachidonic acid (AA) promotes tumorigenesis. Mechanistically, feeding AA enriches gram‐negative bacteria, which promotes the conversion of AA to PGE2 and ultimately promotes CRC development.^193^ Gamma amino butyric acid (GABA) is an inhibitory neurotransmitter expressed in enteric nerves, immune cells, and endocrine‐like cells.^194^, ^195^ GABA is involved in the regulation of gastrointestinal motility.^196^ Activation of GABA receptors inhibits autophagy and enhances the immune response to prevent bacterial infection.^197^ Additionally, GABA receptors have been found to be upregulated in the intestinal epithelium after treatment with 5‐FU, irradiation, and DSS.^194^, ^198^ Both endogenous and exogenous GABA exacerbate intestinal injury by activating GABA receptors.^194^ GABA regulates macrophages and participates in immune regulation. Supplementation with exogenous GABA promotes colon tumor growth. Mechanistically, GABA may facilitate the differentiation of monocytes into anti‐inflammatory macrophages that secrete IL‐10, consequently inhibiting the cytotoxic function of CD8 T cells.^199^

### 4.4 The role of microbial metabolite in gut homeostasis

Metabolites produced by the gut microbiota are key molecular mediators in the crosstalk between the microbiota and the intestine (Table 3). These microbial metabolites can be broadly classified into two categories. The first category includes compounds produced by bacteria that may induce toxic responses in the host, such as LPS and bacterial endotoxins. The second category consists of two subgroups of metabolites associated with the gut microbiota. The first subgroup comprises metabolites generated by intestinal bacteria from dietary components, including short‐chain fatty acids (SCFAs), tryptophan, and indole derivatives. The second subgroup involves metabolites initially produced by the host and subsequently modified by intestinal bacteria, such as bile acids.^200^ LPS enhances apoptosis through the modulation of Toll‐like receptor 4 (TLR4), leading to a reduction in the proliferation of stem and progenitor cells.^201^, ^202^ *Clostridioides difficile* exerts its deleterious effects on colonic stem cells via the exotoxin TcdB, impairing the colon's capacity to repair damaged.^203^ SCFAs, such as acetate, butyrate, and propionate, are metabolites produced through microbial fermentation in the gut.^204^ SCFAs are capable of activating GPR43 in immune cells, thereby playing a significant role in the resolution of inflammation.^205^ Butyrate suppresses the proliferation of stem cells exposed to the intestinal lumen by acetylating histones and modulating the activity of FOXO3.^206^ Tryptophan and its metabolites play a crucial role in regulating intestinal homeostasis, particularly under pathological conditions. Elevated concentrations of tryptophan activate AhR, thereby inhibiting intestinal tumorigenesis through the regulation of E3 ubiquitin ligases ring finger protein 43 (RNF43) and zinc and ring finger 3 (ZNRF3), which suppress WNT/β‐catenin signaling to prevent the overproliferation of ISCs.^207^ Additionally, indole‐3‐aldehyde stimulates lamina propria LPLs to secrete IL‐22 via AhR activation, which subsequently induces STAT3 phosphorylation, accelerating the proliferation of intestinal epithelial cells and promoting the restoration of damaged intestinal mucosa.^208^ Indoleacrylic acid (IA) and indolepropionic acid (IPA) contribute to the protection of the intestinal epithelial barrier by enhancing macrophage function and modulating the inflammatory response, partially through pregnane X receptor (PXR) signaling.^209^, ^210^ Polyamines are small molecules derived from the metabolism of L‐arginine, and the addition of spermidine (SPMD) resulted in the transformation of naive T cells from differentiation into Th17.^211^ The intestine is rich in bile acids, and bile is involved in various physiological processes through various receptors such as farnesoid X receptor (FXR), PXR, and vitamin D receptor (VDR).^212^ Bile extract or lithocholic acid (LCA) promotes intestinal organoid growth by activating G‐protein‐coupled bile acid receptor (TGR5) in the ISC, and LCA inhibits nuclear factor kappa B (NF‐κB) signaling and activates SIRT1/Nrf2 by activating VDR, thereby increasing tight junction proteins and strengthening the epithelial barrier.^213^, ^214^ However, due to the vast variety of metabolites, many remain unknown and unidentified. In the future, advanced approaches in metabolic research are expected to identify more metabolites involved in regulating intestinal homeostasis.

**TABLE 3:** Effects of metabolites on intestinal stem cells (ISC) self‐renewal and intestinal homeostasis.

Metabolites	Regulation of the intestine	Refs.
Lactate	Lactate promotes organoid growth and enhances intestinal immunity.	^114^
β‐OHB	β‐OHB increases the number of Lgr5^+^ ISCs and improves the integrity and survival of the crypts after irradiation.	^143^
Gln	Gln supplementation activates the WNT signaling pathway and self‐renewal of ISCs, and significantly alleviates the intestinal damage caused by burning.	^181^, ^182^
Taurine	Taurine accelerates regeneration of ISCs, promotes intestinal peristalsis, and accelerates glucose metabolism in middle‐aged and elderly mice after supplementation with taurine.	^183^
SAM	Lack of Met in organoid medium inhibits stem cell proliferation in intestinal organoids.	^184^
5‐HT	5‐HT activates PGE2 production in a PGE2 macrophage, promotes WNT/β‐catenin signaling to promote self‐renewal in ISCs.	^185^
α‐KG	Modulating macrophage polarization alleviates DSS‐induced colitis and suppresses tumor growth in colon cancer.	^187^
XANA/KYNA	Supplementation with XANA or KYNA decreases colitis severity through effects on intestinal epithelial cells and T cells.	^215^
GABA	B cell‐derived GABA promotes monocyte differentiation into anti‐inflammatory macrophages that secrete interleukin‐10 and inhibit CD8 T cell killer function.	^199^
LPS	LPS enhances apoptosis through the modulation of TLR4, leading to a reduction in the proliferation of stem and progenitor cells.	^201^, ^202^
TcdB	Exotoxin TcdB impairs the colon's capacity to repair damaged.	^203^
SCFAs	SCFAs activate GPR43 in immune cells and play a significant role in the resolution of inflammation.	^205^
Butyrate	Butyrate suppresses the proliferation of stem cells exposed to the intestinal lumen.	^206^
Tryptophan	Tryptophan suppresses WNT/β‐catenin signaling to prevent the overproliferation of ISCs.	^207^
Indole‐3‐aldehyde	Indole‐3‐aldehyde accelerates the proliferation of intestinal epithelial cells by IL‐22 and STAT3.	^208^
Indoleacrylic acid and indolepropionic acid	They enhance macrophage function and modulates the inflammatory response, partially through PXR signaling.	^209^, ^210^
SPMD	SPMD causes the transformation of naive T cells from differentiation into Th17.	^211^
LCA	LCA promotes intestinal organoid growth by activating TGR5.	^213^, ^214^

Abbreviations: α‐KG, alpha‐ketoglutarate; β‐OHB, beta‐hydroxybutyrate; DSS, dextran sulfate sodium salt; GABA, gamma amino butyric acid; IL‐22, interleukin‐22; ISC, intestinal stem cell; KYNA, kynurenic acid; LCA, lithocholic acid; LPS, lipopolysaccharides; PGE2, prostaglandin E2; PXR, pregnane X receptor; SAM, S‐adenosylmethionine; SCFA, short‐chain fatty acid; SPMD, spermidine; STAT3, signal transducer and activator of transcription 3; TGR5, G‐protein‐coupled bile acid receptor; Th17, T helper cells 17; TLR4, Toll‐like receptor 4; WNT, wingless‐related integration site; XANA, xanthurenic acid; 5‐HT, serotonin.

## 5 FUTURE PERSPECTIVES: ADVANCED APPROACHES IN METABOLIC RESEARCH

### 5.1 Untargeted and targeted metabolomics

The maintenance of intestinal homeostasis is inextricably linked to the dynamic metabolic processes of enterocytes and the intestinal microbiota.^139^ Dietary intake includes approximately 8000 non‐nutritive compounds, such as dietary fiber, and polyphenols.^216^ Most of these compounds cannot be digested by human digestive enzymes and are instead catabolized by gut microbiota, leading to the production of a wide array of metabolites.^217^ The metabolic processes and pathways involved are highly dynamic. Metabolomics, the comprehensive analysis of metabolites in tissues and cells, allows for the detection of subtle changes in biological pathways. This analysis provides critical insights into the mechanisms underlying various physiological conditions and pathological processes. Moreover, metabolomics can be utilized to track metabolites produced by both the host and gut microbes, thereby enhancing our understanding of the complex metabolic interactions between gut microbiota and their hosts.^218^ Metabolites are predominantly characterized using both untargeted and targeted mass spectrometry (MS)‐based metabolomics approaches. Untargeted metabolomics aims to detect the dynamic changes of metabolites within a sample, followed by biostatistical analysis to identify differential metabolites. Targeted metabolomic focuses on the qualitative and quantitative analysis of a specific class of metabolites of interest. Most analyses of fecal metabolomics can be conducted with targeted metabolomics approaches.^219^, ^220^ Liquid chromatography (LC) and gas chromatography (GC) coupled with MS are among the most widely employed platforms in metabolomics research.^221^, ^222^, ^223^, ^224^ LC–MS and GC–MS have different application scenarios. LC–MS is highly sensitive and excels in the separation of thousands of small molecules, including polar metabolites such as organic acids, organic amines, nucleosides, nucleotides, and polyamines. GC–MS is particularly effective for fractionating less polar small molecules, such as alkyl derivatives, AAs, essential oils, esters, fragrances, terpenes, waxes, volatiles, carotenoids, flavonoids, and lipids.^221^ In addition, GC–MS usually requires the derivatization of certain substances through alkylation, acylation, or silylation reactions to enable analysis.

### 5.2 Advances in metabolomics technologies

In fact, there are some challenges and advances in the application of metabolomics. First, both untargeted and targeted metabolomics do not provide information on intracellular metabolic rates and the relative activity of metabolic pathways. The most effective method for elucidating the dynamic metabolic status is metabolic flux analysis (MFA), which involves the use of stable isotopes, such as carbon‐13 (^13^C), nitrogen‐15 (^15^N), or deuterium (^2^H), for tracing purposes.^225^ Isotopic labeling of specific compounds, including nutrients or substrates, allows for the detailed tracking of their metabolic fate within the body.^226^, ^227^ Using ^13^C_16_‐palmitate and ^13^C_2_‐acetate, we followed the metabolic flux and found that loss of HNF4 in small intestinal organoids resulted in impaired TCA cycle but increased fatty acid synthesis.^83^ Second, some metabolites may remain undetectable due to their low abundance or poor ionization efficiency. Derivatization can alter the properties of metabolites and enhance detection sensitivity. It can also facilitate the design of subsequent enrichment steps. For example, ketones and aldehydes are particularly challenging to detect and identify with LC–MS. Conway et al. developed a chemoselective probe immobilized on magnetic beads to improve the metabolites concentration and ionization efficiency. They successfully identified 112 ketone and aldehyde metabolites, and elucidated their exact chemical structures.^228^ Third, the identification of metabolites typically depends on database searches, which can result in the neglect of many unknown metabolites. The focus of microbiome research is increasingly shifting from the gut microbiota to their small molecules.^229^ However, a large number of microbiome‐derived metabolites are not recorded in databases. To address this, researchers have proposed a new approach to metabolomics research—reverse metabolomics. It first requires newly synthesized compounds, followed by LC–MS/MS to obtain the mass spectra of the compounds. Then researchers utilized the Mass Spectrometry Search Tool (MASST) program to search the obtained MS spectra within the existing MS datasets and explored associations between metabolites and various phenotypes, species, and sample types. Using this approach, researchers have identified that bile acids bound to amino acids, such as glutamate, isoleucine/leucine, phenylalanine, threonine, tryptophan, and tyrosine, are elevated in the feces of patients with CD. Additionally, they discovered that cholestatic amide compounds produce by *Bifidobacterium*, *Clostridium*, and *Enterococcus* may contribute to intestinal inflammation by modulating IFNγ production and activating the PXR in T cells.^230^

### 5.3 Future directions in metabolomics research: Single‐cell and spatial levels of metabolomics

Currently, using untargeted and targeted metabolomics techniques can obtain information at the bulk tissue level. However, these methods are limited in their ability to track dynamic metabolism at the single‐cell and spatial levels. For example, stem and progenitor cells within the intestinal epithelium are relatively scarce, but understanding their metabolic characteristics at the single‐cell level is crucial for studying intestinal homeostasis or disease pathogenesis. Metabolomics analysis typically requires millions of cells.^231^ Consequently, the use of small‐scale metabolomics to analyze metabolic changes within cellular subpopulations and achieve spatially resolved metabolic profiling represents a critical direction for future research in metabolomics. Recently, researchers have employed flow cytometry in conjunction with hydrophilic interaction liquid chromatography (HILIC) and high‐sensitivity Orbitrap MS. A total of 160 metabolites were identified using this advanced analytical technique in 10,000 cells.^232^ Additionally, hyperpolarized micromagnetic resonance spectroscopy (HMRS) technology has been developed by integrating nuclear magnetic resonance (NMR) spectroscopy and imaging with hyperpolarization of nuclear spins. This method allows for real‐time metabolic analysis in intact cells or organs and requires only approximately 10,000 cells.^232^ Furthermore, a class of metabolite‐specific biosensors has been developed, which can be combined with single‐cell techniques to dynamically monitor changes in specific metabolites at the single‐cell level. Wu et al. developed a lactate biosensor. When paired with single‐cell imaging, it enables the measurement of glycolytic metabolites. The lactate, glucose, pyruvate, and ATP can be detected in individual endothelial cells.^233^ Similarly, Tao et al. designed a genetically encoded fluorescent indicator for nicotinamide adenine dinucleotide phosphate (NADPH). It is capable of quantifying cytosolic and mitochondrial NADPH pools in a single living cell.^234^

Matrix‐assisted laser desorption/ionization‐mass spectrometry imaging (MALDI‐MSI) enables spatial localization of target metabolites and proteins within tissues.^229^, ^235^ Recently, researchers have developed SpaceM, a technique based on MALDI imaging MS combined with optical microscopy. This method utilizes optical microscopy for image segmentation and calibration, followed by MALDI imaging to analyze metabolites. SpaceM offers nontargeted metabolic profiles, fluorescence intensity measurements, and spatial morphological characteristics at the single‐cell level.^236^ For example, MALDI‐MSI has been employed to detect and spatially map 42 lipid species produced by *Bacteroides thetaiotaomicron* in the mouse colon.^237^ Furthermore, combining MALDI‐MSI with stable isotope tracing allows for the assessment and visualization of region‐specific metabolism. ^13^C‐labeled glycerol and glucose were used to directly visualize glycolytic and gluconeogenic activities in distinct regions of the mouse kidney.^235^ Additionally, to study metabolic differences within organelles, techniques such as organelle precipitation enrichment have been developed. Mitochondrial immunopurification (MITO IP) enables the quantification of metabolites specifically within mitochondria,^238^ and this approach has now been extended to lysosomes and peroxisomes.^239^, ^240^ In summary, single‐cell and spatial levels of metabolomics show tantalizing promise, but large‐scale applications have yet to be realized.

## 6 CONCLUSIONS AND PERSPECTIVES

Extensive research has underscored the pivotal role of the tissue microenvironment in sustaining intestinal homeostasis. This microenvironment comprises mesenchymal cells, immune cells, bacteria, enteric neuronal cells, extracellular matrix, and nutritional factors, all of which interact synergistically to create a milieu conducive to regeneration. The homing capacity of intestinal epithelial cells during tissue repair is intricately regulated by this microenvironment. However, the direct link between the microenvironment and cellular plasticity remains largely unexplored in most studies. Therefore, it is possible to coculture nonepithelial components such as immune cells, bacteria, or fibroblasts with intestinal organoids. It provides an approach to elucidate how the regenerative processes of the gut are perceived and initiated. Future research should prioritize the identification of additional microenvironmental components that facilitate the regeneration of intestinal epithelial cells.

Due to the complex and diverse nature of the gut microbiota, there is a significant gap in understanding the detailed interactions between the gut and ISCs. Although current research on the role of gut microbiota in maintaining homeostasis has primarily concentrated on two commensal species, *Lactobacillus* and *Bifidobacterium*, many questions remain unanswered. Specifically, which other microbial species are involved in the regulation of intestinal homeostasis, and what are the precise mechanisms underlying these regulatory processes? These questions require further investigation. Fecal microbiota transplantation (FMT) has demonstrated significant potential in treating gastrointestinal disorders. However, variations in FMT approaches, including differences in dosage and routes of administration, have led to heterogeneous clinical outcomes.^241^ Consequently, there is an increasing emphasis on optimizing FMT through the development of more precise and effective delivery systems, alongside the formulation of personalized therapeutic strategies tailored to the specific needs of individual patients.

Our review enumerates the various metabolic pathways and metabolites that modulate the function of ISCs and maintain intestinal homeostasis through distinct mechanisms in both physiological and pathological states. In the future, targeting specific metabolic pathways and metabolites may emerge as a novel therapeutic strategy for intestinal diseases. Additionally, dietary restriction and the supplementation of specific metabolites hold promise as innovative forms of adjuvant therapy. However, current metabolic analyses predominantly focus on bulk tissue levels. Understanding the dynamic metabolic processes of ISCs and progenitor cells is crucial. Furthermore, recent research on gut microbiota has increasingly shifted from examining the microbiota itself to investigating the metabolites it produces, a transition made possible by the advent of advanced metabolomic techniques. In conclusion, achieving spatially resolved metabolism is a critical direction for future research in metabolomics.

## AUTHOR CONTRIBUTIONS

Lei Chen developed the initial idea, conceived, and supervised the study. Ruolan Zhang wrote the initial draft of the manuscript; Ansu Perekatt edited the draft. Lei Chen revised and approved the final draft. All the authors have read and approved the final manuscript.

## CONFLICT OF INTEREST STATEMENT

The authors declare no conflicts of interest.

## ETHICS STATEMENT

Not applicable.

## Data Availability

Not applicable.

## References

[1] Beumer J , Clevers H . Cell fate specification and differentiation in the adult mammalian intestine. Nat Rev Mol Cell Biol. 2021;22(1):39‐53. 32958874 10.1038/s41580-020-0278-0

[2] Colozza G , Park S‐Y , Koo B‐K . Clone wars: From molecules to cell competition in intestinal stem cell homeostasis and disease. Exp Mol Med. 2022;54(9):1367‐1378. 36117218 10.1038/s12276-022-00854-5 PMC9534868

[3] Chen L , Qiu X , Dupre A , et al. TGFB1 induces fetal reprogramming and enhances intestinal regeneration. Cell Stem Cell. 2023;30(11):1520‐1537.e8. 37865088 10.1016/j.stem.2023.09.015 PMC10841757

[4] Jang J , Jeong S . Inflammatory bowel disease: pathophysiology, treatment, and disease modeling. BioChip J. 2023;17(4):403‐430.

[5] Cheng Z , Wang T , Jiao Y , et al. Burden of digestive system diseases in China and its provinces during 1990–2019: results of the 2019 Global Disease Burden Study. Chin Med J (Engl). 2024; 137(18):2182‐2189. 39138597 10.1097/CM9.0000000000003277 PMC11407821

[6] Ma T , Wan M , Liu G , Zuo X , Yang X , Yang X . Temporal trends of inflammatory bowel disease burden in China from 1990 to 2030 with comparisons to Japan, South Korea, the European Union, the United States of America, and the world. Clin Epidemiol. 2023;15:583‐599. 37187768 10.2147/CLEP.S402718 PMC10178411

[7] Kiela PR , Ghishan FK . Physiology of intestinal absorption and secretion. Best Pract Res Clin Gastroenterol. 2016;30(2):145‐159. 27086882 10.1016/j.bpg.2016.02.007 PMC4956471

[8] Snoeck V , Goddeeris B , Cox E . The role of enterocytes in the intestinal barrier function and antigen uptake. Microbes Infect. 2005;7(7–8):997‐1004. 15925533 10.1016/j.micinf.2005.04.003

[9] Haber AL , Biton M , Rogel N , et al. A single‐cell survey of the small intestinal epithelium. Nature. 2017;551(7680):333‐339. 29144463 10.1038/nature24489 PMC6022292

[10] Muñoz J , Stange DE , Schepers AG , et al. The Lgr5 intestinal stem cell signature: robust expression of proposed quiescent ‘+4’ cell markers. EMBO J. 2012;31(14):3079‐3091. 22692129 10.1038/emboj.2012.166 PMC3400017

[11] van der Flier LG , van Gijn ME , Hatzis P , et al. Transcription factor achaete scute‐like 2 controls intestinal stem cell fate. Cell. 2009;136(5):903‐912. 19269367 10.1016/j.cell.2009.01.031

[12] van der Flier LG , Haegebarth A , Stange DE , van de Wetering M , Clevers H . OLFM4 is a robust marker for stem cells in human intestine and marks a subset of colorectal cancer cells. Gastroenterology. 2009;137(1):15‐17. 19450592 10.1053/j.gastro.2009.05.035

[13] Montgomery RK , Carlone DL , Richmond CA , et al. Mouse telomerase reverse transcriptase (mTert) expression marks slowly cycling intestinal stem cells. Proc Natl Acad Sci USA. 2011;108(1):179‐184. 21173232 10.1073/pnas.1013004108 PMC3017192

[14] Capdevila C , Miller J , Cheng L , et al. Time‐resolved fate mapping identifies the intestinal upper crypt zone as an origin of *Lgr5*+ crypt base columnar cells. Cell. 2024;187(12):3039‐3055.e14. 38848677 10.1016/j.cell.2024.05.001 PMC11770878

[15] Schuijers J , Junker Jan P , Mokry M , et al. Ascl2 acts as an R‐spondin/Wnt‐responsive switch to control stemness in intestinal crypts. Cell Stem Cell. 2015;16(2):158‐170. 25620640 10.1016/j.stem.2014.12.006

[16] Yui S , Azzolin L , Maimets M , et al. YAP/TAZ‐dependent reprogramming of colonic epithelium links ECM remodeling to tissue regeneration. Cell Stem Cell. 2018;22(1):35‐49.e7. 29249464 10.1016/j.stem.2017.11.001 PMC5766831

[17] VanDussen KL , Carulli AJ , Keeley TM , et al. Notch signaling modulates proliferation and differentiation of intestinal crypt base columnar stem cells. Development. 2012;139(3):488‐497. 22190634 10.1242/dev.070763 PMC3252352

[18] Kosinski C , Li VSW , Chan ASY , et al. Gene expression patterns of human colon tops and basal crypts and BMP antagonists as intestinal stem cell niche factors. Proc Natl Acad Sci. 2007;104(39):15418‐15423. 17881565 10.1073/pnas.0707210104 PMC2000506

[19] Fre S , Huyghe M , Mourikis P , Robine S , Louvard D , Artavanis‐Tsakonas S . Notch signals control the fate of immature progenitor cells in the intestine. Nature. 2005;435(7044):964‐968. 15959516 10.1038/nature03589

[20] Sato T , Vries RG , Snippert HJ , et al. Single Lgr5 stem cells build crypt‐villus structures in vitro without a mesenchymal niche. Nature. 2009;459(7244):262‐265. 19329995 10.1038/nature07935

[21] Kolev HM , Kaestner KH . Mammalian intestinal development and differentiation—the state of the art. Cell Mol Gastroenterol Hepatol. 2023;16(5):809‐821. 37507088 10.1016/j.jcmgh.2023.07.011 PMC10520362

[22] Zorn AM , Wells JM . Vertebrate endoderm development and organ formation. Annu Rev Cell Dev Biol. 2009;25:221‐251. 19575677 10.1146/annurev.cellbio.042308.113344 PMC2861293

[23] Chin AM , Hill DR , Aurora M , Spence JR . Morphogenesis and maturation of the embryonic and postnatal intestine. Semin Cell Dev Biol. 2017;66:81‐93. 28161556 10.1016/j.semcdb.2017.01.011 PMC5487846

[24] Shyer AE , Huycke TR , Lee C , Mahadevan L , Tabin CJ . Bending gradients: how the intestinal stem cell gets its home. Cell. 2015;161(3):569‐580. 25865482 10.1016/j.cell.2015.03.041 PMC4409931

[25] Fordham Robert P , Yui S , Hannan Nicholas RF , et al. Transplantation of expanded fetal intestinal progenitors contributes to colon regeneration after injury. Cell Stem Cell. 2013;13(6):734‐744. 24139758 10.1016/j.stem.2013.09.015 PMC3858813

[26] Ayyaz A , Kumar S , Sangiorgi B , et al. Single‐cell transcriptomes of the regenerating intestine reveal a revival stem cell. Nature. 2019;569(7754):121‐125. 31019301 10.1038/s41586-019-1154-y

[27] Gehart H , Clevers H . Tales from the crypt: new insights into intestinal stem cells. Nat Rev Gastroenterol Hepatol. 2019;16(1):19‐34. 30429586 10.1038/s41575-018-0081-y

[28] Hageman JH , Heinz MC , Kretzschmar K , van der Vaart J , Clevers H , Snippert HJG . Intestinal regeneration: regulation by the microenvironment. Dev Cell. 2020;54(4):435‐446. 32841594 10.1016/j.devcel.2020.07.009

[29] Gayer CP , Basson MD . The effects of mechanical forces on intestinal physiology and pathology. Cell Signal. 2009;21(8):1237‐1244. 19249356 10.1016/j.cellsig.2009.02.011 PMC2715958

[30] Fernández‐Sánchez ME , Barbier S , Whitehead J , et al. Mechanical induction of the tumorigenic β‐catenin pathway by tumour growth pressure. Nature. 2015;523(7558):92‐95. 25970250 10.1038/nature14329

[31] de Sousa EMF , de Sauvage FJ . Cellular plasticity in intestinal homeostasis and disease. Cell Stem Cell. 2019;24(1):54‐64. 30595498 10.1016/j.stem.2018.11.019

[32] Palikuqi B , Rispal J , Reyes EA , Vaka D , Boffelli D , Klein O . Lymphangiocrine signals are required for proper intestinal repair after cytotoxic injury. Cell Stem Cell. 2022;29(8):1262‐1272.e5. 35931034 10.1016/j.stem.2022.07.007 PMC9387209

[33] Akpolat M , Oz ZS , Gulle K , Hamamcioglu AC , Bakkal BH , Kececi M . X irradiation induced colonic mucosal injury and the detection of apoptosis through PARP‐1/p53 regulatory pathway. Biomed Pharmacother. 2020;127:110134. 32361637 10.1016/j.biopha.2020.110134

[34] Zhou W‐J , Geng ZH , Spence JR , Geng J‐G . Induction of intestinal stem cells by R‐spondin 1 and Slit2 augments chemoradioprotection. Nature. 2013;501(7465):107‐111. 23903657 10.1038/nature12416 PMC3888063

[35] Jalili‐Firoozinezhad S , Prantil‐Baun R , Jiang A , et al. Modeling radiation injury‐induced cell death and countermeasure drug responses in a human gut‐on‐a‐chip. Cell Death Dis. 2018;9(2):223. 29445080 10.1038/s41419-018-0304-8 PMC5833800

[36] Leonetti D , Estéphan H , Ripoche N , et al. Secretion of acid sphingomyelinase and ceramide by endothelial cells contributes to radiation‐induced intestinal toxicity. Cancer Res. 2020;80(12):2651‐2662. 32291318 10.1158/0008-5472.CAN-19-1527

[37] Malipatlolla DK , Patel P , Sjöberg F , et al. Long‐term mucosal injury and repair in a murine model of pelvic radiotherapy. Sci Rep. 2019;9(1):13803. 31551503 10.1038/s41598-019-50023-4 PMC6760522

[38] Blirando K , Milliat F , Martelly I , Sabourin JC , Benderitter M , François A . Mast cells are an essential component of human radiation proctitis and contribute to experimental colorectal damage in mice. Am J Pathol. 2011;178(2):640‐651. 21281796 10.1016/j.ajpath.2010.10.003 PMC3069878

[39] Schmitt M , Schewe M , Sacchetti A , et al. Paneth cells respond to inflammation and contribute to tissue regeneration by acquiring stem‐like features through SCF/c‐kit signaling. Cell Rep. 2018;24(9):2312‐2328.e7. 30157426 10.1016/j.celrep.2018.07.085

[40] Xin J‐Y , Wang J , Ding Q‐Q , et al. Potential role of gut microbiota and its metabolites in radiation‐induced intestinal damage. Ecotoxicol Environ Saf. 2022;248:114341. 36442401 10.1016/j.ecoenv.2022.114341

[41] Andersson‐Rolf A , Zilbauer M , Koo BK , Clevers H . Stem cells in repair of gastrointestinal epithelia. Physiology (Bethesda). 2017;32(4):278‐289. 28615312 10.1152/physiol.00005.2017 PMC5545610

[42] Miyoshi H , Ajima R , Luo CT , Yamaguchi TP , Stappenbeck TS . Wnt5a potentiates TGF‐β signaling to promote colonic crypt regeneration after tissue injury. Science. 2012;338(6103):108‐113. 22956684 10.1126/science.1223821 PMC3706630

[43] Metcalfe C , Kljavin NM , Ybarra R , de Sauvage FJ . Lgr5+ stem cells are indispensable for radiation‐induced intestinal regeneration. Cell Stem Cell. 2014;14(2):149‐159. 24332836 10.1016/j.stem.2013.11.008

[44] van Es JH , Sato T , van de Wetering M , et al. Dll1+ secretory progenitor cells revert to stem cells upon crypt damage. Nat Cell Biol. 2012;14(10):1099‐1104. 23000963 10.1038/ncb2581 PMC3789123

[45] Castillo‐Azofeifa D , Fazio EN , Nattiv R , et al. Atoh1(+) secretory progenitors possess renewal capacity independent of Lgr5(+) cells during colonic regeneration. EMBO J. 2019;38(4):e99984. 30635334 10.15252/embj.201899984 PMC6376326

[46] Yu S , Tong K , Zhao Y , et al. Paneth cell multipotency induced by notch activation following Injury. Cell Stem Cell. 2018;23(1):46‐59.e5. 29887318 10.1016/j.stem.2018.05.002 PMC6035085

[47] Powell AE , Wang Y , Li Y , et al. The pan‐ErbB negative regulator Lrig1 is an intestinal stem cell marker that functions as a tumor suppressor. Cell. 2012;149(1):146‐158. 22464327 10.1016/j.cell.2012.02.042 PMC3563328

[48] Malagola E , Vasciaveo A , Ochiai Y , et al. Isthmus progenitor cells contribute to homeostatic cellular turnover and support regeneration following intestinal injury. Cell. 2024;187(12):3056‐3071.e17. 38848678 10.1016/j.cell.2024.05.004 PMC11164536

[49] Harnack C , Berger H , Antanaviciute A , et al. R‐spondin 3 promotes stem cell recovery and epithelial regeneration in the colon. Nat Commun. 2019;10(1):4368. 31554819 10.1038/s41467-019-12349-5 PMC6761174

[50] Tian H , Biehs B , Warming S , et al. A reserve stem cell population in small intestine renders Lgr5‐positive cells dispensable. Nature. 2011;478(7368):255‐259. 21927002 10.1038/nature10408 PMC4251967

[51] Yousefi M , Li N , Nakauka‐Ddamba A , et al. Msi RNA‐binding proteins control reserve intestinal stem cell quiescence. J Cell Biol. 2016;215(3):401‐413. 27799368 10.1083/jcb.201604119 PMC5100293

[52] Sangiorgi E , Capecchi MR . Bmi1 is expressed in vivo in intestinal stem cells. Nat Genet. 2008;40(7):915‐920. 18536716 10.1038/ng.165 PMC2906135

[53] Yan KS , Gevaert O , Zheng GXY , et al. Intestinal enteroendocrine lineage cells possess homeostatic and injury‐inducible stem cell activity. Cell Stem Cell. 2017;21(1):78‐90.e6. 28686870 10.1016/j.stem.2017.06.014 PMC5642297

[54] Barker N . Adult intestinal stem cells: critical drivers of epithelial homeostasis and regeneration. Nat Rev Mol Cell Biol. 2014;15(1):19‐33. 24326621 10.1038/nrm3721

[55] Wong VWY , Stange DE , Page ME , et al. Lrig1 controls intestinal stem‐cell homeostasis by negative regulation of ErbB signalling. Nat Cell Biol. 2012;14(4):401‐408. 22388892 10.1038/ncb2464 PMC3378643

[56] Powell Anne E , Wang Y , Li Y , et al. The Pan‐ErbB negative regulator Lrig1 is an intestinal stem cell marker that functions as a tumor suppressor. Cell. 2012;149(1):146‐158. 22464327 10.1016/j.cell.2012.02.042 PMC3563328

[57] Nusse YM , Savage AK , Marangoni P , et al. Parasitic helminths induce fetal‐like reversion in the intestinal stem cell niche. Nature. 2018;559(7712):109‐113. 29950724 10.1038/s41586-018-0257-1 PMC6042247

[58] Kabiri Z , Greicius G , Madan B , et al. Stroma provides an intestinal stem cell niche in the absence of epithelial Wnts. Development. 2014;141(11):2206‐2215. 24821987 10.1242/dev.104976

[59] Johansson J , Naszai M , Hodder MC , et al. RAL GTPases drive intestinal stem cell function and regeneration through internalization of WNT signalosomes. Cell Stem Cell. 2019;24(4):592‐607.e7. 30853556 10.1016/j.stem.2019.02.002 PMC6459002

[60] Okamoto R , Tsuchiya K , Nemoto Y , et al. Requirement of notch activation during regeneration of the intestinal epithelia. Am J Physiol Gastrointest Liver Physiol. 2009;296(1):G23‐G35. 19023031 10.1152/ajpgi.90225.2008

[61] Gregorieff A , Liu Y , Inanlou MR , Khomchuk Y , Wrana JL . Yap‐dependent reprogramming of Lgr5(+) stem cells drives intestinal regeneration and cancer. Nature. 2015;526(7575):715‐718. 26503053 10.1038/nature15382

[62] Pikkupeura LM , Bressan RB , Guiu J , et al. Transcriptional and epigenomic profiling identifies YAP signaling as a key regulator of intestinal epithelium maturation. Sci Adv. 2023;9(28):eadf9460. 37436997 10.1126/sciadv.adf9460 PMC10337905

[63] Serra D , Mayr U , Boni A , et al. Self‐organization and symmetry breaking in intestinal organoid development. Nature. 2019;569(7754):66‐72. 31019299 10.1038/s41586-019-1146-y PMC6544541

[64] Drozdowski L , Thomson AB . Aging and the intestine. World J Gastroenterol. 2006;12(47):7578‐7584. 17171784 10.3748/wjg.v12.i47.7578 PMC4088037

[65] Vazquez Roque M , Bouras EP . Epidemiology and management of chronic constipation in elderly patients. Clin Interv Aging. 2015;10:919‐930. 26082622 10.2147/CIA.S54304 PMC4459612

[66] Dekker E , Tanis PJ , Vleugels JLA , Kasi PM , Wallace MB . Colorectal cancer. Lancet. 2019;394(10207):1467‐1480. 31631858 10.1016/S0140-6736(19)32319-0

[67] Funk MC , Gleixner JG , Heigwer F , et al. Aged intestinal stem cells propagate cell‐intrinsic sources of inflammaging in mice. Dev Cell. 2023;58(24):2914‐2929.e7. 38113852 10.1016/j.devcel.2023.11.013

[68] Funk MC , Zhou J , Boutros M . Ageing, metabolism and the intestine. EMBO Rep. 2020;21(7):e50047. 32567155 10.15252/embr.202050047 PMC7332987

[69] Moorefield EC , Andres SF , Blue RE , et al. Aging effects on intestinal homeostasis associated with expansion and dysfunction of intestinal epithelial stem cells. Aging (Albany N Y). 2017;9(8):1898‐1915. 10.18632/aging.101279 PMC5611984 28854151

[70] Nalapareddy K , Nattamai KJ , Kumar RS , et al. Canonical Wnt signaling ameliorates aging of intestinal stem cells. Cell Rep. 2017;18(11):2608‐2621. 28297666 10.1016/j.celrep.2017.02.056 PMC5987258

[71] Omrani O , Krepelova A , Rasa SMM , et al. IFNγ‐Stat1 axis drives aging‐associated loss of intestinal tissue homeostasis and regeneration. Nat Commun. 2023;14(1):6109. 37777550 10.1038/s41467-023-41683-y PMC10542816

[72] Igarashi M , Miura M , Williams E , et al. NAD+ supplementation rejuvenates aged gut adult stem cells. Aging Cell. 2019;18(3):e12935. 30917412 10.1111/acel.12935 PMC6516145

[73] Mihaylova MM , Cheng C‐W , Cao AQ , et al. Fasting activates fatty acid oxidation to enhance intestinal stem cell function during homeostasis and aging. Cell Stem Cell. 2018;22(5):769‐778.e4. 29727683 10.1016/j.stem.2018.04.001 PMC5940005

[74] Pentinmikko N , Iqbal S , Mana M , et al. Notum produced by Paneth cells attenuates regeneration of aged intestinal epithelium. Nature. 2019;571(7765):398‐402. 31292548 10.1038/s41586-019-1383-0 PMC8151802

[75] Nefzger CM , Jardé T , Srivastava A , et al. Intestinal stem cell aging signature reveals a reprogramming strategy to enhance regenerative potential. NPJ Regen Med. 2022;7(1):31. 35710627 10.1038/s41536-022-00226-7 PMC9203768

[76] Yang L , Ruan Z , Lin X , et al. NAD+ dependent UPRmt activation underlies intestinal aging caused by mitochondrial DNA mutations. Nat Commun. 2024;15(1):546. 38228611 10.1038/s41467-024-44808-z PMC10791663

[77] He D , Wu H , Xiang J , et al. Gut stem cell aging is driven by mTORC1 via a p38 MAPK‐p53 pathway. Nat Commun. 2020;11(1):37. 31896747 10.1038/s41467-019-13911-x PMC6940394

[78] Yun J , Hansen S , Morris O , et al. Senescent cells perturb intestinal stem cell differentiation through Ptk7 induced noncanonical Wnt and YAP signaling. Nat Commun. 2023;14(1):156. 36631445 10.1038/s41467-022-35487-9 PMC9834240

[79] Luissint AC , Parkos CA , Nusrat A . Inflammation and the intestinal barrier: leukocyte‐epithelial cell interactions, cell junction remodeling, and mucosal repair. Gastroenterology. 2016;151(4):616‐632. 27436072 10.1053/j.gastro.2016.07.008 PMC5317033

[80] Ren W‐y , Wu K‐f , Li X , et al. Age‐related changes in small intestinal mucosa epithelium architecture and epithelial tight junction in rat models. Aging Clin Exp Res. 2014;26(2):183‐191. 24243034 10.1007/s40520-013-0148-0

[81] Kühn F , Adiliaghdam F , Cavallaro PM , et al. Intestinal alkaline phosphatase targets the gut barrier to prevent aging. JCI Insight. 2020;5(6):e134049. 32213701 10.1172/jci.insight.134049 PMC7213802

[82] Shen X , Gao X , Luo Y , et al. Cxxc finger protein 1 maintains homeostasis and function of intestinal group 3 innate lymphoid cells with aging. Nature Aging. 2023;3(8):965‐981. 37429951 10.1038/s43587-023-00453-7

[83] Chen L , Vasoya RP , Toke NH , et al. HNF4 regulates fatty acid oxidation and is required for renewal of intestinal stem cells in mice. Gastroenterology. 2020;158(4):985‐999.e9. 31759926 10.1053/j.gastro.2019.11.031 PMC7062567

[84] Gu W , Wang H , Huang X , et al. SATB2 preserves colon stem cell identity and mediates ileum‐colon conversion via enhancer remodeling. Cell Stem Cell. 2022;29(1):101‐115.e10. 34582804 10.1016/j.stem.2021.09.004 PMC8741647

[85] Chen L , Toke NH , Luo S , et al. HNF4 factors control chromatin accessibility and are redundantly required for maturation of the fetal intestine. Development. 2019;146(19):dev179432. 31345929 10.1242/dev.179432 PMC6803367

[86] Kim C‐K , Saxena M , Maharjan K , et al. Krüppel‐like factor 5 regulates stemness, lineage specification, and regeneration of intestinal epithelial stem cells. Cell Mol Gastroenterol Hepatol. 2020;9(4):587‐609. 31778829 10.1016/j.jcmgh.2019.11.009 PMC7078555

[87] Jardé T , Chan WH , Rossello FJ , et al. Mesenchymal niche‐derived neuregulin‐1 drives intestinal stem cell proliferation and regeneration of damaged epithelium. Cell Stem Cell. 2020;27(4):646‐662.e7. 32693086 10.1016/j.stem.2020.06.021

[88] Roulis M , Kaklamanos A , Schernthanner M , et al. Paracrine orchestration of intestinal tumorigenesis by a mesenchymal niche. Nature. 2020;580(7804):524‐529. 32322056 10.1038/s41586-020-2166-3 PMC7490650

[89] Martín‐Alonso M , Iqbal S , Vornewald PM , et al. Smooth muscle‐specific MMP17 (MT4‐MMP) regulates the intestinal stem cell niche and regeneration after damage. Nat Commun. 2021;12(1):6741. 34795242 10.1038/s41467-021-26904-6 PMC8602650

[90] Kim TH , Saadatpour A , Guo G , et al. Single‐cell transcript profiles reveal multilineage priming in early progenitors derived from Lgr5(+) intestinal stem cells. Cell Rep. 2016;16(8):2053‐2060. 27524622 10.1016/j.celrep.2016.07.056 PMC5001892

[91] Ritsma L , Ellenbroek SIJ , Zomer A , et al. Intestinal crypt homeostasis revealed at single‐stem‐cell level by in vivo live imaging. Nature. 2014;507(7492):362‐365. 24531760 10.1038/nature12972 PMC3964820

[92] Krndija D , El Marjou F , Guirao B , et al. Active cell migration is critical for steady‐state epithelial turnover in the gut. Science. 2019;365(6454):705‐710. 31416964 10.1126/science.aau3429

[93] Merlos‐Suárez A , Barriga Francisco M , Jung P , et al. The intestinal stem cell signature identifies colorectal cancer stem cells and predicts disease relapse. Cell Stem Cell. 2011;8(5):511‐524. 21419747 10.1016/j.stem.2011.02.020

[94] Chen L , Cao W , Aita R , et al. Three‐dimensional interactions between enhancers and promoters during intestinal differentiation depend upon HNF4. Cell Rep. 2021;34(4):108679. 33503426 10.1016/j.celrep.2020.108679 PMC7899294

[95] Chen L , Luo S , Dupre A , et al. The nuclear receptor HNF4 drives a brush border gene program conserved across murine intestine, kidney, and embryonic yolk sac. Nat Commun. 2021;12(1):2886. 34001900 10.1038/s41467-021-22761-5 PMC8129143

[96] Chen L , Toke NH , Luo S , et al. A reinforcing HNF4–SMAD4 feed‐forward module stabilizes enterocyte identity. Nat Genet. 2019;51(5):777‐785. 30988513 10.1038/s41588-019-0384-0 PMC6650150

[97] Silberg DG , Sullivan J , Kang E , et al. Cdx2 ectopic expression induces gastric intestinal metaplasia in transgenic mice. Gastroenterology. 2002;122(3):689‐696. 11875002 10.1053/gast.2002.31902

[98] Gao N , White P , Kaestner KH . Establishment of intestinal identity and epithelial‐mesenchymal signaling by *Cdx2* . Dev Cell. 2009;16(4):588‐599. 19386267 10.1016/j.devcel.2009.02.010 PMC2673200

[99] Säisä‐Borreill S , Davidson G , Kleiber T , et al. General transcription factor TAF4 antagonizes epigenetic silencing by polycomb to maintain intestine stem cell functions. Cell Death Differ. 2023;30(3):839‐853. 36639541 10.1038/s41418-022-01109-6 PMC9984434

[100] Rao‐Bhatia A , Zhu M , Yin W‐C , et al. Hedgehog‐activated Fat4 and PCP pathways mediate mesenchymal cell clustering and villus formation in gut development. Dev Cell. 2020;52(5):647‐658.e6. 32155439 10.1016/j.devcel.2020.02.003

[101] Gu W , Huang X , Singh PNP , et al. A MTA2‐SATB2 chromatin complex restrains colonic plasticity toward small intestine by retaining HNF4A at colonic chromatin. Nat Commun. 2024;15(1):3595. 38678016 10.1038/s41467-024-47738-y PMC11055869

[102] Swisa A , Kieckhaefer J , Daniel SG , et al. The evolutionarily ancient FOXA transcription factors shape the murine gut microbiome via control of epithelial glycosylation. Dev Cell. 2024;59(16):2069‐2084.e8. 38821056 10.1016/j.devcel.2024.05.006 PMC11338728

[103] Wu N , Sun H , Zhao X , et al. MAP3K2‐regulated intestinal stromal cells define a distinct stem cell niche. Nature. 2021;592(7855):606‐610. 33658717 10.1038/s41586-021-03283-y

[104] Roulis M , Flavell RA . Fibroblasts and myofibroblasts of the intestinal lamina propria in physiology and disease. Differentiation. 2016;92(3):116‐131. 27165847 10.1016/j.diff.2016.05.002

[105] Greicius G , Kabiri Z , Sigmundsson K , et al. PDGFRα(+) pericryptal stromal cells are the critical source of Wnts and RSPO3 for murine intestinal stem cells in vivo. Proc Natl Acad Sci USA. 2018;115(14):E3173‐e3181. 29559533 10.1073/pnas.1713510115 PMC5889626

[106] Beumer J , Puschhof J , Bauzá‐Martinez J , et al. High‐resolution mRNA and secretome atlas of human enteroendocrine cells. Cell. 2020;181(6):1291‐1306.e19. 32407674 10.1016/j.cell.2020.04.036

[107] Beumer J , Artegiani B , Post Y , et al. Enteroendocrine cells switch hormone expression along the crypt‐to‐villus BMP signalling gradient. Nat Cell Biol. 2018;20(8):909‐916. 30038251 10.1038/s41556-018-0143-y PMC6276989

[108] Holloway EM , Czerwinski M , Tsai YH , et al. Mapping development of the human intestinal niche at single‐cell resolution. Cell Stem Cell. 2021;28(3):568‐580.e4. 33278341 10.1016/j.stem.2020.11.008 PMC7935765

[109] Goto N , Goto S , Imada S , Hosseini S , Deshpande V , Yilmaz ÖH . Lymphatics and fibroblasts support intestinal stem cells in homeostasis and injury. Cell Stem Cell. 2022;29(8):1246‐1261.e6. 35931033 10.1016/j.stem.2022.06.013 PMC9720889

[110] Giri J , Das R , Nylen E , Chinnadurai R , Galipeau J . CCL2 and CXCL12 derived from mesenchymal stromal cells cooperatively polarize IL‐10+ tissue macrophages to mitigate gut injury. Cell Rep. 2020;30(6):1923‐1934.e4. 32049021 10.1016/j.celrep.2020.01.047 PMC7043065

[111] Wang X , Cai J , Lin B , et al. GPR34‐mediated sensing of lysophosphatidylserine released by apoptotic neutrophils activates type 3 innate lymphoid cells to mediate tissue repair. Immunity. 2021;54(6):1123‐1136.e8. 34107271 10.1016/j.immuni.2021.05.007

[112] Wu H , Xie S , Miao J , et al. *Lactobacillus reuteri* maintains intestinal epithelial regeneration and repairs damaged intestinal mucosa. Gut Microbes. 2020;11(4):997‐1014. 32138622 10.1080/19490976.2020.1734423 PMC7524370

[113] Danhof HA , Lee J , Thapa A , Britton RA , Di Rienzi SC . Microbial stimulation of oxytocin release from the intestinal epithelium via secretin signaling. Gut Microbes. 2023;15(2):2256043. 37698879 10.1080/19490976.2023.2256043 PMC10498800

[114] Lee Y‐S , Kim T‐Y , Kim Y , et al. Microbiota‐derived lactate accelerates intestinal stem‐cell‐mediated epithelial development. Cell Host Microbe. 2018;24(6):833‐846.e6. 30543778 10.1016/j.chom.2018.11.002

[115] Bhattarai Y , Williams BB , Battaglioli EJ , et al. Gut microbiota‐produced tryptamine activates an epithelial G‐protein‐coupled receptor to increase colonic secretion. Cell Host Microbe. 2018;23(6):775‐785.e5. 29902441 10.1016/j.chom.2018.05.004 PMC6055526

[116] Hu J , Chen J , Xu X , Hou Q , Ren J , Yan X . Gut microbiota‐derived 3‐phenylpropionic acid promotes intestinal epithelial barrier function via AhR signaling. Microbiome. 2023;11(1):102. 37158970 10.1186/s40168-023-01551-9 PMC10165798

[117] Martinez‐Guryn K , Hubert N , Frazier K , et al. Small intestine microbiota regulate host digestive and absorptive adaptive responses to dietary lipids. Cell Host Microbe. 2018;23(4):458‐469.e5. 29649441 10.1016/j.chom.2018.03.011 PMC5912695

[118] Gong T , Liu L , Jiang W , Zhou R . DAMP‐sensing receptors in sterile inflammation and inflammatory diseases. Nat Rev Immunol. 2020;20(2):95‐112. 31558839 10.1038/s41577-019-0215-7

[119] Eming SA , Wynn TA , Martin P . Inflammation and metabolism in tissue repair and regeneration. Science. 2017;356(6342):1026‐1030. 28596335 10.1126/science.aam7928

[120] Gronke K , Diefenbach A . Regenerative biology: innate immunity repairs gut lining. Nature. 2015;528(7583):488‐489. 26649826 10.1038/nature16325

[121] Wang P , Kljavin N , Nguyen TTT , et al. Adrenergic nerves regulate intestinal regeneration through IL‐22 signaling from type 3 innate lymphoid cells. Cell Stem Cell. 2023;30(9):1166‐1178.e8. 37597516 10.1016/j.stem.2023.07.013

[122] Neil JA , Matsuzawa‐Ishimoto Y , Kernbauer‐Hölzl E , et al. IFN‐I and IL‐22 mediate protective effects of intestinal viral infection. Nat Microbiol. 2019;4(10):1737‐1749. 31182797 10.1038/s41564-019-0470-1 PMC6871771

[123] Zmora N , Suez J , Elinav E . You are what you eat: diet, health and the gut microbiota. Nat Rev Gastroenterol Hepatol. 2019;16(1):35‐56. 30262901 10.1038/s41575-018-0061-2

[124] Liu X , Nagy P , Bonfini A , et al. Microbes affect gut epithelial cell composition through immune‐dependent regulation of intestinal stem cell differentiation. Cell Rep. 2022;38(13):110572. 35354023 10.1016/j.celrep.2022.110572 PMC9078081

[125] Fulde M , Sommer F , Chassaing B , et al. Neonatal selection by Toll‐like receptor 5 influences long‐term gut microbiota composition. Nature. 2018;560(7719):489‐493. 30089902 10.1038/s41586-018-0395-5

[126] Sommer F , Bäckhed F . The gut microbiota—masters of host development and physiology. Nat Rev Microbiol. 2013;11(4):227‐238. 23435359 10.1038/nrmicro2974

[127] O'Hara AM , Shanahan F . The gut flora as a forgotten organ. EMBO Rep. 2006;7(7):688‐693. 16819463 10.1038/sj.embor.7400731 PMC1500832

[128] Hooper LV . Bacterial contributions to mammalian gut development. Trends Microbiol. 2004;12(3):129‐134. 15001189 10.1016/j.tim.2004.01.001

[129] Abo H , Chassaing B , Harusato A , et al. Erythroid differentiation regulator‐1 induced by microbiota in early life drives intestinal stem cell proliferation and regeneration. Nat Commun. 2020;11(1):513. 31980634 10.1038/s41467-019-14258-z PMC6981263

[130] Ma N , Chen X , Johnston LJ , Ma X . Gut microbiota‐stem cell niche crosstalk: a new territory for maintaining intestinal homeostasis. iMeta. 2022;1(4):e54. 38867904 10.1002/imt2.54 PMC10989768

[131] Liu C , Ma N , Feng Y , et al. From probiotics to postbiotics: concepts and applications. Anim Res One Health. 2023;1(1):92‐114.

[132] Kim HJ , Kim YJ , Kim YJ , et al. Microbiota influences host exercise capacity via modulation of skeletal muscle glucose metabolism in mice. Exp Mol Med. 2023;55(8):1820‐1830. 37542180 10.1038/s12276-023-01063-4 PMC10474268

[133] Perekatt AO , Valdez MJ , Davila M , et al. YY1 is indispensable for Lgr5^+^ intestinal stem cell renewal. Proc Natl Acad Sci. 2014;111(21):7695‐7700. 24821761 10.1073/pnas.1400128111 PMC4040551

[134] Kumar N , Srivillibhuthur M , Joshi S , et al. A YY1‐dependent increase in aerobic metabolism is indispensable for intestinal organogenesis. Development. 2016;143(20):3711‐3722. 27802136 10.1242/dev.137992 PMC5087649

[135] Ludikhuize MC , Meerlo M , Gallego MP , et al. Mitochondria define intestinal stem cell differentiation downstream of a FOXO/notch axis. Cell Metab. 2020;32(5):889‐900.e7. 33147486 10.1016/j.cmet.2020.10.005

[136] Berger E , Rath E , Yuan D , et al. Mitochondrial function controls intestinal epithelial stemness and proliferation. Nat Commun. 2016;7(1):13171. 27786175 10.1038/ncomms13171 PMC5080445

[137] Khaloian S , Rath E , Hammoudi N , et al. Mitochondrial impairment drives intestinal stem cell transition into dysfunctional Paneth cells predicting Crohn's disease recurrence. Gut. 2020;69(11):1939‐1951. 32111634 10.1136/gutjnl-2019-319514 PMC7569388

[138] Moschandrea C , Kondylis V , Evangelakos I , et al. Mitochondrial dysfunction abrogates dietary lipid processing in enterocytes. Nature. 2024;625(7994):385‐392. 38123683 10.1038/s41586-023-06857-0 PMC10781618

[139] Ulgherait M , Chen A , McAllister SF , et al. Circadian regulation of mitochondrial uncoupling and lifespan. Nat Commun. 2020;11(1):1927. 32317636 10.1038/s41467-020-15617-x PMC7174288

[140] Li C , Zhou Y , Wei R , et al. Glycolytic regulation of intestinal stem cell self‐renewal and differentiation. Cell Mol Gastroenterol Hepatol. 2023;15(4):931‐947. 36584817 10.1016/j.jcmgh.2022.12.012 PMC9971054

[141] Schell JC , Wisidagama DR , Bensard C , et al. Control of intestinal stem cell function and proliferation by mitochondrial pyruvate metabolism. Nat Cell Biol. 2017;19(9):1027‐1036. 28812582 10.1038/ncb3593 PMC6137334

[142] Stine RR , Sakers AP , TeSlaa T , et al. PRDM16 maintains homeostasis of the intestinal epithelium by controlling region‐specific metabolism. Cell Stem Cell. 2019;25(6):830‐845.e8. 31564549 10.1016/j.stem.2019.08.017 PMC6898778

[143] Cheng C‐W , Biton M , Haber AL , et al. Ketone body signaling mediates intestinal stem cell homeostasis and adaptation to diet. Cell. 2019;178(5):1115‐1131.e15. 31442404 10.1016/j.cell.2019.07.048 PMC6732196

[144] Wei X , Yang Z , Rey Federico E , et al. Fatty acid synthase modulates intestinal barrier function through palmitoylation of mucin 2. Cell Host Microbe. 2012;11(2):140‐152. 22341463 10.1016/j.chom.2011.12.006 PMC3285413

[145] Li S , Lu C‐W , Diem EC , et al. Acetyl‐CoA‐carboxylase 1‐mediated de novo fatty acid synthesis sustains Lgr5+ intestinal stem cell function. Nat Commun. 2022;13(1):3998. 35810180 10.1038/s41467-022-31725-2 PMC9271096

[146] Setiawan J , Kotani T , Konno T , et al. Regulation of small intestinal epithelial homeostasis by Tsc2‐mTORC1 signaling. Kobe J Med Sci. 2019;64(6):E200‐e209. 31327863 PMC6668652

[147] Najumudeen AK , Ceteci F , Fey SK , et al. The amino acid transporter SLC7A5 is required for efficient growth of KRAS‐mutant colorectal cancer. Nat Genet. 2021;53(1):16‐26. 33414552 10.1038/s41588-020-00753-3

[148] Wong CC , Qian Y , Li X , et al. SLC25A22 promotes proliferation and survival of colorectal cancer cells with KRAS mutations and xenograft tumor progression in mice via intracellular synthesis of aspartate. Gastroenterology. 2016;151(5):945‐960.e6. 27451147 10.1053/j.gastro.2016.07.011

[149] Tajan M , Hock AK , Blagih J , et al. A role for p53 in the adaptation to glutamine starvation through the expression of SLC1A3. Cell Metab. 2018;28(5):721‐736.e6. 30122553 10.1016/j.cmet.2018.07.005 PMC6224545

[150] Calibasi‐Kocal G , Mashinchian O , Basbinar Y , Ellidokuz E , Cheng C‐W , Yilmaz ÖH . Nutritional control of intestinal stem cells in homeostasis and tumorigenesis. Trends Endocrinol Metab. 2021;32(1):20‐35. 33277157 10.1016/j.tem.2020.11.003

[151] Yilmaz ÖH , Katajisto P , Lamming DW , et al. mTORC1 in the Paneth cell niche couples intestinal stem‐cell function to calorie intake. Nature. 2012;486(7404):490‐495. 22722868 10.1038/nature11163 PMC3387287

[152] Liu Y , Yang K , Jia Y , et al. Gut microbiome alterations in high‐fat‐diet‐fed mice are associated with antibiotic tolerance. Nat Microbiol. 2021;6(7):874‐884. 34017107 10.1038/s41564-021-00912-0

[153] Fan Z , Zhang X , Shang Y , et al. Intestinal flora changes induced by a high‐fat diet promote activation of primordial follicles through macrophage infiltration and inflammatory factor secretion in mouse ovaries. Int J Mol Sci. 2022;23(9):4797. 35563189 10.3390/ijms23094797 PMC9100959

[154] Fontana L , Partridge L . Promoting health and longevity through diet: from model organisms to humans. Cell. 2015;161(1):106‐118. 25815989 10.1016/j.cell.2015.02.020 PMC4547605

[155] Fontana L , Mitchell SE , Wang B , et al. The effects of graded caloric restriction: XII. Comparison of mouse to human impact on cellular senescence in the colon. Aging Cell. 2018;17(3):e12746. 29575469 10.1111/acel.12746 PMC5946078

[156] Cui J , Shi S , Sun X , et al. Mitochondrial autophagy involving renal injury and aging is modulated by caloric intake in aged rat kidneys. PLoS ONE. 2013;8(7):e69720. 23894530 10.1371/journal.pone.0069720 PMC3718786

[157] Catterson JH , Khericha M , Dyson MC , et al. Short‐term, intermittent fasting induces long‐lasting gut health and TOR‐independent lifespan extension. Curr Biol. 2018;28(11):1714‐1724.e4. 29779873 10.1016/j.cub.2018.04.015 PMC5988561

[158] Beyaz S , Mana MD , Roper J , et al. High‐fat diet enhances stemness and tumorigenicity of intestinal progenitors. Nature. 2016;531(7592):53‐58. 26935695 10.1038/nature17173 PMC4846772

[159] Ang QY , Alexander M , Newman JC , et al. Ketogenic diets alter the gut microbiome resulting in decreased intestinal Th17 cells. Cell. 2020;181(6):1263‐1275.e16. 32437658 10.1016/j.cell.2020.04.027 PMC7293577

[160] Tang F‐Y , Pai M‐H , Chiang E‐PI . Consumption of high‐fat diet induces tumor progression and epithelial–mesenchymal transition of colorectal cancer in a mouse xenograft model. J Nutr Biochem. 2012;23(10):1302‐1313. 22221675 10.1016/j.jnutbio.2011.07.011

[161] Goncalves MD , Lu C , Tutnauer J , et al. High‐fructose corn syrup enhances intestinal tumor growth in mice. Science. 2019;363(6433):1345‐1349. 30898933 10.1126/science.aat8515 PMC6487857

[162] Rodríguez‐Colman MJ , Schewe M , Meerlo M , et al. Interplay between metabolic identities in the intestinal crypt supports stem cell function. Nature. 2017;543(7645):424‐427. 28273069 10.1038/nature21673

[163] Yang L , Ruan Z , Lin X , et al. NAD(+) dependent UPR(mt) activation underlies intestinal aging caused by mitochondrial DNA mutations. Nat Commun. 2024;15(1):546. 38228611 10.1038/s41467-024-44808-z PMC10791663

[164] Trifunovic A , Wredenberg A , Falkenberg M , et al. Premature ageing in mice expressing defective mitochondrial DNA polymerase. Nature. 2004;429(6990):417‐423. 15164064 10.1038/nature02517

[165] Passos JF , Nelson G , Wang C , et al. Feedback between p21 and reactive oxygen production is necessary for cell senescence. Mol Syst Biol. 2010;6:347. 20160708 10.1038/msb.2010.5 PMC2835567

[166] Velarde MC , Flynn JM , Day NU , Melov S , Campisi J . Mitochondrial oxidative stress caused by Sod2 deficiency promotes cellular senescence and aging phenotypes in the skin. Aging (Albany N Y). 2012;4(1):3‐12. 10.18632/aging.100423 PMC3292901 22278880

[167] Wiley CD , Velarde MC , Lecot P , et al. Mitochondrial dysfunction induces senescence with a distinct secretory phenotype. Cell Metab. 2016;23(2):303‐314. 26686024 10.1016/j.cmet.2015.11.011 PMC4749409

[168] Ito K , Carracedo A , Weiss D , et al. A PML–PPAR‐δ pathway for fatty acid oxidation regulates hematopoietic stem cell maintenance. Nat Med. 2012;18(9):1350‐1358. 22902876 10.1038/nm.2882 PMC3566224

[169] Stoll EA , Makin R , Sweet IR , et al. Neural stem cells in the adult subventricular zone oxidize fatty acids to produce energy and support neurogenic activity. Stem Cells. 2015;33(7):2306‐2319. 25919237 10.1002/stem.2042 PMC4478223

[170] Ducheix S , Peres C , Härdfeldt J , et al. Deletion of stearoyl‐CoA desaturase‐1 from the intestinal epithelium promotes inflammation and tumorigenesis, reversed by dietary oleate. Gastroenterology. 2018;155(5):1524‐1538.e9. 30063922 10.1053/j.gastro.2018.07.032

[171] Flor AC , Wolfgeher D , Wu D , Kron SJ . A signature of enhanced lipid metabolism, lipid peroxidation and aldehyde stress in therapy‐induced senescence. Cell Death Discov. 2017;3:17075. 29090099 10.1038/cddiscovery.2017.75 PMC5661608

[172] Wiley CD , Sharma R , Davis SS , et al. Oxylipin biosynthesis reinforces cellular senescence and allows detection of senolysis. Cell Metab. 2021;33(6):1124‐1136.e5. 33811820 10.1016/j.cmet.2021.03.008 PMC8501892

[173] Wiley CD , Campisi J . The metabolic roots of senescence: mechanisms and opportunities for intervention. Nat Metab. 2021;3(10):1290‐1301. 34663974 10.1038/s42255-021-00483-8 PMC8889622

[174] Tsugawa H , Ishihara T , Ogasa K , et al. A lipidome landscape of aging in mice. Nat Aging. 2024;4(5):709‐726. 38609525 10.1038/s43587-024-00610-6

[175] Ling Z‐N , Jiang Y‐F , Ru J‐N , Lu J‐H , Ding B , Wu J . Amino acid metabolism in health and disease. Signal Transd Targeted Ther. 2023;8(1):345. 10.1038/s41392-023-01569-3 PMC10497558 37699892

[176] Barron L , Sun RC , Aladegbami B , Erwin CR , Warner BW , Guo J . Intestinal epithelial‐specific mTORC1 activation enhances intestinal adaptation after small bowel resection. Cell Mol Gastroenterol Hepatol. 2017;3(2):231‐244. 28275690 10.1016/j.jcmgh.2016.10.006 PMC5331783

[177] Xiang L , Mou J , Shao B , et al. Glutaminase 1 expression in colorectal cancer cells is induced by hypoxia and required for tumor growth, invasion, and metastatic colonization. Cell Death Dis. 2019;10(2):40. 30674873 10.1038/s41419-018-1291-5 PMC6426853

[178] Deng L , Yao P , Li L , et al. p53‐mediated control of aspartate‐asparagine homeostasis dictates LKB1 activity and modulates cell survival. Nat Commun. 2020;11(1):1755. 32273511 10.1038/s41467-020-15573-6 PMC7145870

[179] Muthusamy T , Cordes T , Handzlik MK , et al. Serine restriction alters sphingolipid diversity to constrain tumour growth. Nature. 2020;586(7831):790‐795. 32788725 10.1038/s41586-020-2609-x PMC7606299

[180] Han YM , Bedarida T , Ding Y , et al. β‐Hydroxybutyrate prevents vascular senescence through hnRNP A1‐mediated upregulation of Oct4. Mol Cell. 2018;71(6):1064‐1078.e5. 30197300 10.1016/j.molcel.2018.07.036 PMC6230553

[181] Tian J , Li Y , Bao X , et al. Glutamine boosts intestinal stem cell‐mediated small intestinal epithelial development during early weaning: involvement of WNT signaling. Stem Cell Rep. 2023;18(7):1451‐1467. 10.1016/j.stemcr.2023.05.012 PMC10362502 37327782

[182] Chen X , Zhang P , Zhang Y , et al. Potential effect of glutamine in the improvement of intestinal stem cell proliferation and the alleviation of burn‐induced intestinal injury via activating YAP: a preliminary study. Nutrients. 2023;15(7):1766. 37049605 10.3390/nu15071766 PMC10097377

[183] Singh P , Gollapalli K , Mangiola S , et al. Taurine deficiency as a driver of aging. Science. 2023;380(6649):eabn9257. 37289866 10.1126/science.abn9257 PMC10630957

[184] Li M‐L , Cao S‐Y , Qu J , et al. S‐adenosyl‐L‐methionine supplementation alleviates damaged intestinal epithelium and inflammatory infiltration caused by Mat2a deficiency. Development. 2023;150(20):dev201135. 36975381 10.1242/dev.201135

[185] Zhu P , Lu T , Wu J , et al. Gut microbiota drives macrophage‐dependent self‐renewal of intestinal stem cells via niche enteric serotonergic neurons. Cell Res. 2022;32(6):555‐569. 35379903 10.1038/s41422-022-00645-7 PMC9160288

[186] Morita N , Umemoto E , Fujita S , et al. GPR31‐dependent dendrite protrusion of intestinal CX3CR1+ cells by bacterial metabolites. Nature. 2019;566(7742):110‐114. 30675063 10.1038/s41586-019-0884-1

[187] Zhang F‐L , Hu Z , Wang Y‐F , et al. Organoids transplantation attenuates intestinal ischemia/reperfusion injury in mice through L‐malic acid‐mediated M2 macrophage polarization. Nat Commun. 2023;14(1):6779. 37880227 10.1038/s41467-023-42502-0 PMC10600233

[188] Tian Q , Bravo Iniguez A , Sun Q , Wang H , Du M , Zhu M‐J . Dietary alpha‐ketoglutarate promotes epithelial metabolic transition and protects against DSS‐induced colitis. Mol Nutr Food Res. 2021;65(7):2000936. 10.1002/mnfr.202000936 33547710

[189] Kozar RA , Schultz SG , Bick RJ , Poindexter BJ , DeSoignie R , Moore FA . Enteral glutamine but not alanine maintains small bowel barrier function after ischemia/reperfusion injury in rats. Shock. 2004;21(5):433‐437. 15087819 10.1097/00024382-200405000-00006

[190] Seth A , Basuroy S , Sheth P , Rao RK . L‐glutamine ameliorates acetaldehyde‐induced increase in paracellular permeability in Caco‐2 cell monolayer. Am J Physiol‐Gastrointest Liver Physiol. 2004;287(3):G510‐G517. 15331350 10.1152/ajpgi.00058.2004

[191] Chloé M , Camille D , Allison A , et al. Rewiring the altered tryptophan metabolism as a novel therapeutic strategy in inflammatory bowel diseases. Gut. 2023;72(7):1296. 36270778 10.1136/gutjnl-2022-327337 PMC10314090

[192] Tran TQ , Hanse EA , Habowski AN , et al. α‐Ketoglutarate attenuates Wnt signaling and drives differentiation in colorectal cancer. Nat Cancer. 2020;1(3):345‐358. 32832918 10.1038/s43018-020-0035-5 PMC7442208

[193] Xu C , Gu L , Hu L , et al. FADS1‐arachidonic acid axis enhances arachidonic acid metabolism by altering intestinal microecology in colorectal cancer. Nat Commun. 2023;14(1):2042. 37041160 10.1038/s41467-023-37590-x PMC10090135

[194] Ma X , Sun Q , Sun X , et al. Activation of GABA(A) receptors in colon epithelium exacerbates acute colitis. Front Immunol. 2018;9:987. 29867964 10.3389/fimmu.2018.00987 PMC5949344

[195] Hyland NP , Cryan JF . A gut feeling about GABA: focus on GABA(B) receptors. Front Pharmacol. 2010;1:124. 21833169 10.3389/fphar.2010.00124 PMC3153004

[196] Auteri M , Zizzo MG , Serio R . GABA and GABA receptors in the gastrointestinal tract: from motility to inflammation. Pharmacol Res. 2015;93:11‐21. 25526825 10.1016/j.phrs.2014.12.001

[197] Kim JK , Kim YS , Lee H‐M , et al. GABAergic signaling linked to autophagy enhances host protection against intracellular bacterial infections. Nat Commun. 2018;9(1):4184. 30305619 10.1038/s41467-018-06487-5 PMC6180030

[198] Zhang C , Zhou Y , Zheng J , et al. Inhibition of GABAA receptors in intestinal stem cells prevents chemoradiotherapy‐induced intestinal toxicity. J Exp Med. 2022;219(12):e20220541. 36125780 10.1084/jem.20220541 PMC9499828

[199] Zhang B , Vogelzang A , Miyajima M , et al. B cell‐derived GABA elicits IL‐10+ macrophages to limit anti‐tumour immunity. Nature. 2021;599(7885):471‐476. 34732892 10.1038/s41586-021-04082-1 PMC8599023

[200] Krautkramer KA , Fan J , Bäckhed F . Gut microbial metabolites as multi‐kingdom intermediates. Nat Rev Microbiol. 2021;19(2):77‐94. 32968241 10.1038/s41579-020-0438-4

[201] Santaolalla R , Sussman DA , Ruiz JR , et al. TLR4 activates the β‐catenin pathway to cause intestinal neoplasia. PLoS ONE. 2013;8(5):e63298. 23691015 10.1371/journal.pone.0063298 PMC3653932

[202] Neal MD , Sodhi CP , Jia H , et al. Toll‐like receptor 4 is expressed on intestinal stem cells and regulates their proliferation and apoptosis via the p53 up‐regulated modulator of apoptosis. J Biol Chem. 2012;287(44):37296‐37308. 22955282 10.1074/jbc.M112.375881 PMC3481327

[203] Mileto SJ , Jardé T , Childress KO , et al. *Clostridioides difficile* infection damages colonic stem cells via TcdB, impairing epithelial repair and recovery from disease. Proc Natl Acad Sci USA. 2020;117(14):8064‐8073. 32198200 10.1073/pnas.1915255117 PMC7149309

[204] Agus A , Clément K , Sokol H . Gut microbiota‐derived metabolites as central regulators in metabolic disorders. Gut. 2021;70(6):1174‐1182. 33272977 10.1136/gutjnl-2020-323071 PMC8108286

[205] Maslowski KM , Vieira AT , Ng A , et al. Regulation of inflammatory responses by gut microbiota and chemoattractant receptor GPR43. Nature. 2009;461(7268):1282‐1286. 19865172 10.1038/nature08530 PMC3256734

[206] Kaiko GE , Ryu SH , Koues OI , et al. The colonic crypt protects stem cells from microbiota‐derived metabolites. Cell. 2016;165(7):1708‐1720. 27264604 10.1016/j.cell.2016.05.018 PMC5026192

[207] Metidji A , Omenetti S , Crotta S , et al. The environmental sensor AHR protects from inflammatory damage by maintaining intestinal stem cell homeostasis and barrier integrity. Immunity. 2018;49(2):353‐362.e5. 30119997 10.1016/j.immuni.2018.07.010 PMC6104739

[208] Hou Q , Ye L , Liu H , et al. *Lactobacillus* accelerates ISCs regeneration to protect the integrity of intestinal mucosa through activation of STAT3 signaling pathway induced by LPLs secretion of IL‐22. Cell Death Differ. 2018;25(9):1657‐1670. 29459771 10.1038/s41418-018-0070-2 PMC6143595

[209] Wlodarska M , Luo C , Kolde R , et al. Indoleacrylic acid produced by commensal *Peptostreptococcus* species suppresses inflammation. Cell Host Microbe. 2017;22(1):25‐37.e6. 28704649 10.1016/j.chom.2017.06.007 PMC5672633

[210] Venkatesh M , Mukherjee S , Wang H , et al. Symbiotic bacterial metabolites regulate gastrointestinal barrier function via the xenobiotic sensor PXR and Toll‐like receptor 4. Immunity. 2014;41(2):296‐310. 25065623 10.1016/j.immuni.2014.06.014 PMC4142105

[211] Carriche GM , Almeida L , Stüve P , et al. Regulating T‐cell differentiation through the polyamine spermidine. J Allergy Clin Immunol. 2021;147(1):335‐348.e11. 32407834 10.1016/j.jaci.2020.04.037

[212] Wahlström A , Sayin SI , Marschall HU , Bäckhed F . Intestinal crosstalk between bile acids and microbiota and its impact on host metabolism. Cell Metab. 2016;24(1):41‐50. 27320064 10.1016/j.cmet.2016.05.005

[213] Sorrentino G , Perino A , Yildiz E , et al. Bile acids signal via TGR5 to activate intestinal stem cells and epithelial regeneration. Gastroenterology. 2020;159(3):956‐968.e8. 32485177 10.1053/j.gastro.2020.05.067

[214] Yao B , He J , Yin X , Shi Y , Wan J , Tian Z . The protective effect of lithocholic acid on the intestinal epithelial barrier is mediated by the vitamin D receptor via a SIRT1/Nrf2 and NF‐κB dependent mechanism in Caco‐2 cells. Toxicol Lett. 2019;316:109‐118. 31472180 10.1016/j.toxlet.2019.08.024

[215] Michaudel C , Danne C , Agus A , et al. Rewiring the altered tryptophan metabolism as a novel therapeutic strategy in inflammatory bowel diseases. Gut. 2023;72(7):1296‐1307. 36270778 10.1136/gutjnl-2022-327337 PMC10314090

[216] Wishart DS . Metabolomics: applications to food science and nutrition research. Trends Food Sci Technol. 2008;19(9):482‐493.

[217] Lamichhane S , Sen P , Dickens AM , Orešič M , Bertram HC . Gut metabolome meets microbiome: a methodological perspective to understand the relationship between host and microbe. Methods. 2018;149:3‐12. 29715508 10.1016/j.ymeth.2018.04.029

[218] Johnson CH , Ivanisevic J , Siuzdak G . Metabolomics: beyond biomarkers and towards mechanisms. Nat Rev Mol Cell Biol. 2016;17(7):451‐459. 26979502 10.1038/nrm.2016.25 PMC5729912

[219] Griffiths WJ , Sjövall J . Bile acids: analysis in biological fluids and tissues. J Lipid Res. 2010;51(1):23‐41. 20008121 10.1194/jlr.R001941-JLR200 PMC2789783

[220] Han J , Lin K , Sequeira C , Borchers CH . An isotope‐labeled chemical derivatization method for the quantitation of short‐chain fatty acids in human feces by liquid chromatography–tandem mass spectrometry. Anal Chim Acta. 2015;854:86‐94. 25479871 10.1016/j.aca.2014.11.015

[221] Sinem N . Metabolomics: basic principles and strategies. In: Sinem N , Hakima A , eds. Molecular Medicine. IntechOpen; 2019:Ch. 8.

[222] Sun J , Beger RD , Schnackenberg LK . Metabolomics as a tool for personalizing medicine: 2012 update. Per Med. 2013;10(2):149‐161. 29758850 10.2217/pme.13.8

[223] Guijas C , Montenegro‐Burke JR , Warth B , Spilker ME , Siuzdak G . Metabolomics activity screening for identifying metabolites that modulate phenotype. Nat Biotechnol. 2018;36(4):316‐320. 29621222 10.1038/nbt.4101 PMC5937131

[224] Marshall DD , Powers R . Beyond the paradigm: combining mass spectrometry and nuclear magnetic resonance for metabolomics. Prog Nucl Magn Reson Spectrosc. 2017;100:1‐16. 28552170 10.1016/j.pnmrs.2017.01.001 PMC5448308

[225] Hiller K , Metallo C , Stephanopoulos G . Elucidation of cellular metabolism via metabolomics and stable‐isotope assisted metabolomics. Curr Pharm Biotechnol. 2011;12(7):1075‐1086. 21466455 10.2174/138920111795909096

[226] Long CP , Antoniewicz MR . High‐resolution ^13^C metabolic flux analysis. Nat Protoc. 2019;14(10):2856‐2877. 31471597 10.1038/s41596-019-0204-0

[227] Ursell LK , Haiser HJ , Van Treuren W , et al. The intestinal metabolome: an intersection between microbiota and host. Gastroenterology. 2014;146(6):1470‐1476. 24631493 10.1053/j.gastro.2014.03.001 PMC4102302

[228] Conway LP , Garg N , Lin W , Vujasinovic M , Löhr JM , Globisch D . Chemoselective probe for detailed analysis of ketones and aldehydes produced by gut microbiota in human samples. Chem Commun. 2019;55(62):9080‐9083. 10.1039/C9CC04605D 31287110

[229] DeBerardinis RJ , Keshari KR . Metabolic analysis as a driver for discovery, diagnosis, and therapy. Cell. 2022;185(15):2678‐2689. 35839759 10.1016/j.cell.2022.06.029 PMC9469798

[230] Gentry EC , Collins SL , Panitchpakdi M , et al. Reverse metabolomics for the discovery of chemical structures from humans. Nature. 2024;626(7998):419‐426. 38052229 10.1038/s41586-023-06906-8 PMC10849969

[231] Jang C , Chen L , Rabinowitz JD . Metabolomics and isotope tracing. Cell. 2018;173(4):822‐837. 29727671 10.1016/j.cell.2018.03.055 PMC6034115

[232] DeVilbiss AW , Zhao Z , Martin‐Sandoval MS , et al. Metabolomic profiling of rare cell populations isolated by flow cytometry from tissues. eLife. 2021;10:e61980. 33470192 10.7554/eLife.61980 PMC7847306

[233] Wu D , Harrison DL , Szasz T , et al. Single‐cell metabolic imaging reveals a SLC2A3‐dependent glycolytic burst in motile endothelial cells. Nat Metab. 2021;3(5):714‐727. 34031595 10.1038/s42255-021-00390-y PMC8362837

[234] Tao R , Zhao Y , Chu H , et al. Genetically encoded fluorescent sensors reveal dynamic regulation of NADPH metabolism. Nat Methods. 2017;14(7):720‐728. 28581494 10.1038/nmeth.4306 PMC5555402

[235] Wang L , Xing X , Zeng X , et al. Spatially resolved isotope tracing reveals tissue metabolic activity. Nat Methods. 2022;19(2):223‐230. 35132243 10.1038/s41592-021-01378-y PMC10926149

[236] Rappez L , Stadler M , Triana S , et al. SpaceM reveals metabolic states of single cells. Nat Methods. 2021;18(7):799‐805. 34226721 10.1038/s41592-021-01198-0 PMC7611214

[237] Mirretta Barone C , Heaver SL , Gruber L , Zundel F , Vu DL , Ley RE . Spatially resolved lipidomics shows conditional transfer of lipids produced by *Bacteroides thetaiotaomicron* into the mouse gut. Cell Host Microbe. 2024;32(6):1025‐1036.e5. 38795710 10.1016/j.chom.2024.04.021

[238] Chen WW , Freinkman E , Wang T , Birsoy K , Sabatini DM . Absolute quantification of matrix metabolites reveals the dynamics of mitochondrial metabolism. Cell. 2016;166(5):1324‐1337.e11. 27565352 10.1016/j.cell.2016.07.040 PMC5030821

[239] Abu‐Remaileh M , Wyant GA , Kim C , et al. Lysosomal metabolomics reveals V‐ATPase‐ and mTOR‐dependent regulation of amino acid efflux from lysosomes. Science. 2017;358(6364):807‐813. 29074583 10.1126/science.aan6298 PMC5704967

[240] Ray GJ , Boydston EA , Shortt E , et al. A PEROXO‐Tag enables rapid isolation of peroxisomes from human cells. iScience. 2020;23(5):101109. 32417403 10.1016/j.isci.2020.101109 PMC7254474

[241] Schmidt TSB , Li SS , Maistrenko OM , et al. Drivers and determinants of strain dynamics following fecal microbiota transplantation. Nat Med. 2022;28(9):1902‐1912. 36109636 10.1038/s41591-022-01913-0 PMC9499871

